# Concurrent Prebiotic Intake Reverses Insulin Resistance Induced by Early-Life Pulsed Antibiotic in Rats

**DOI:** 10.3390/biomedicines9010066

**Published:** 2021-01-12

**Authors:** Teja Klancic, Isabelle Laforest-Lapointe, Jolene Wong, Ashley Choo, Jodi E. Nettleton, Faye Chleilat, Marie-Claire Arrieta, Raylene A. Reimer

**Affiliations:** 1 Faculty of Kinesiology, University of Calgary, Calgary, AB T2N 1N4, Canada; klancic.teja@gmail.com (T.K.); jolene.wong@ualberta.ca (J.W.); acchoo@ucalgary.ca (A.C.); jenettle@ucalgary.ca (J.E.N.); fatima.chleilat1@ucalgary.ca (F.C.); 2 Department of Physiology & Pharmacology, Cumming School of Medicine, University of Calgary, Calgary, AB T2N 4N1, Canada; Isabelle.Laforest-Lapointe@USherbrooke.ca (I.L.-L.); marie.arrieta@ucalgary.ca (M.-C.A.); 3 Department of Pediatrics, Cumming School of Medicine, University of Calgary, Calgary, AB T2N 4N1, Canada; 4 Department of Biochemistry & Molecular Biology, Cumming School of Medicine, University of Calgary, Calgary, AB T2N 4N1, Canada

**Keywords:** antibiotic, prebiotic, oligofructose, insulin resistance, obesity, gut microbiota

## Abstract

Pulsed antibiotic treatment (PAT) early in life increases risk of obesity. Prebiotics can reduce fat mass and improve metabolic health. We examined if co-administering prebiotic with PAT reduces obesity risk in rat pups weaned onto a high fat/sucrose diet. Pups were randomized to (1) control [CTR], (2) antibiotic [ABT] (azithromycin), (3) prebiotic [PRE] (10% oligofructose (OFS)), (4) antibiotic + prebiotic [ABT + PRE]. Pulses of antibiotics/prebiotics were administered at d19–21, d28–30 and d37–39. Male and female rats given antibiotics (ABT) had higher body weight than all other groups at 10 wk of age. The PAT phenotype was stronger in ABT males than females, where increased fat mass, hyperinsulinemia and insulin resistance were present and all reversible with prebiotics. Reduced hypothalamic and hepatic expression of insulin receptor substrates and ileal tight junction proteins was seen in males only, explaining their greater insulin resistance. In females, insulin resistance was improved with prebiotics and normalized to lean control. ABT reduced *Lactobacillaceae* and increased *Bacteroidaceae* in both sexes. Using a therapeutic dose of an antibiotic commonly used for acute infection in children, PAT increased body weight and impaired insulin production and insulin sensitivity. The effects were reversed with prebiotic co-administration in a sex-specific manner.

## 1. Introduction

The gut microbiota has co-evolved with its human host, conferring a wide-range of metabolic, nutritional, and immunological benefits for the host [1]. However, disruptions to the microbial community (dybiosis) can contribute to obesity and other metabolic diseases [2]. Alterations to the gut microbiota are more likely to occur early in life due to microbial instability and a higher likelihood of exposure to external factors that could cause perturbations such as antibiotics [3]. Antibiotics are one of the most commonly prescribed therapeutic agents around the world [4]. Exposure to antibiotics in early life is of particular concern given that ~90% of exclusively breast-fed infant microbiota are bifidobacteria, which are highly susceptible to antibiotics [5]. Reduced bifidobacteria abundance is found in individuals with higher BMI [6,7] and a negative correlation has been shown between *Bifidobacterium* and visceral adiposity (6). Disrupting gut microbiota during critical developmental windows can have lifetime consequences due to limited “microbial pressure” resulting in abnormal immune system maturation [8,9]. In addition to disrupting gut microbiota development, antibiotics also impair the ability of microbes to deal with stressors such as a high fat, high sugar diet [10].

Decades ago, experiments in farm animals showed that low dose (subtherapeutic) administration of antibiotic promoted growth [11]. More recent experiments in mice showed that early-life sub-therapeutic antibiotic treatment (STAT) increased body weight/fat mass and worsened metabolic outcomes alongside altered gut microbiota [12]. Fecal microbiota transplant showed that the obese phenotype could be transferred from donor to germ free recipient mice demonstrating the important causative role the microbial community plays in early-life antibiotic-induced obesity risk [12]. Importantly, we recently demonstrated that the negative metabolic and phenotypic side effects of STAT were prevented when prebiotic oligofructose was co-administered with antibiotic in pregnant and lactating dams and their offspring [13].

The most recent definition of a prebiotic is “a substrate that is selectively utilized by host microorganisms conferring a health benefit” [14]. Prebiotics, particularly chicory root-derived inulin and oligofructose have been shown in multiple studies in humans and animals to reduce body weight and fat mass, increase serum satiety hormones, reduce inflammation and increase levels of health promoting *Bifidobacterium* [14,15,16,17].

Given the high rates of antibiotic use, particularly in children, there is a need to identify strategies that will attenuate the known risks associated with their exposure in early life. Therefore, our objective was to mimic paediatric antibiotic use and determine whether the negative metabolic outcomes of early life antibiotic use could be mitigated by co-administering prebiotic oligofructose. To mimic human antibiotic treatment, we administered therapeutic doses of azithromycin, a commonly used antibiotic in humans [18] and animals [19]. In a 3-year cohort study involving more than 30 million individuals in the USA, azithromycin was found to be the most commonly prescribed antibiotic between 2013 and 2015 [18]. Importantly, our schedule of administration (10 mg/kg/day for 3 consecutive days) mimicked administration regimen used in children [20]. Key parameters that we investigated were body weight and composition, metabolic hormones (insulin/leptin/GLP-1), longitudinal microbiota changes and hypothalamic/hepatic gene expression.

Our results show increased body weight of males and females given antibiotics early in life with greater fat mass accumulation in males. In addition, antibiotics impaired insulin sensitivity, select hepatic and hypothalamic gene expression as well as microbiota development (reduced *Lactobacillaceae* and increased *Bacteroidaceae* levels). We demonstrate that co-administering prebiotic oligofructose with therapeutic doses of antibiotics prevents the negative metabolic side effects of antibiotics.

## 2. Experimental Section

### 2.1. Animals and Diets

A total of 30 Sprague Dawley rats (10 weeks old, *n* = 20 females, *n* = 10 males) were obtained from Charles River Laboratories (Saint Constant, QC, Canada) and housed individually on a 12 h light–dark cycle in a temperature (20–22 °C) and humidity (41–60%) controlled room. After 2 weeks of acclimatization, rats were mated to generate pups for inclusion in the intervention study. Within 24 h of birth, litters were culled to 10 pups (*n* = 5 males, *n* = 5 females). Pregnant/lactating dams consumed normal chow (Lab Diet 5001, LabDiet, St. Louis, MO, USA) throughout the study. Pups were cross-fostered at 19 days of age (to reduce litter effect) and dams with their newly composed litters were randomized to (*n* = 20 pups/group): (1) control [CTR], (2) antibiotic [ABT] (azithromycin; dose 10 mg/kg/day; oral suspension concentration of 200 mg/5 mL; zithromax; Pfizer (Kirkland, QC, Canada) [21]), (3) prebiotic [PRE] (10% oligofructose (OFS) oral suspension/diet, 10% wt/wt, Orafti P95 (BENEO GmbH, Mannheim, Germany), (4) antibiotic + prebiotic [ABT + PRE] and (5) lean control [LEAN]. The first pulse of antibiotic/prebiotic was administered before weaning from d19–21 of life through a feeding dropper. The second and third pulse of antibiotic were given d28–30 and d37–39, respectively. Animals were weaned onto a high-fat/high sucrose diet (HFS) at d21 (diet #102412; Dyets, Bethlehem, PA, USA), with prebiotic groups (P and AP) consuming 10% OFS in their diet. The diet composition has been previously published [16]. Prebiotic groups remained on the diet until the end of the last pulse of antibiotics. A fifth lean reference group (*n* = 20) was maintained on standard chow diet throughout the study. Animals were euthanized for tissue collection at two time points: wk7–after the last antibiotic pulse and wk10–end of study (*n* = 10 pups/group per time point). The 10% OFS dose was selected based on previous experiments showing reductions in fat mass [22] and increases in *Bifidobacterium* spp. and *Lactobacillus* spp. favoring a lean phenotype [23,24]. The amount of azithromycin given to the pups was calculated based on their body weight. This dose (10 mg/kg/day, three consecutive days, three pulses) and type of antibiotic is therapeutic for rodents and mirrors the concentration commonly used for human children for an acute infection [25]. Ethical approval was granted by the University of Calgary Animal Care Committee (Protocol #: AC15-0079, July 30, 2018) and conformed to the *Guide for the Care and Use of Laboratory Animals*.

### 2.2. Tissue and Blood Collection

Following a 12-h overnight fast, animals were euthanized with over-anesthetization with isoflurane and aortic cut. Tissues (cecum, liver and hypothalamus) from the rats at 7 and 10 weeks of age were weighed, collected and snap frozen in liquid nitrogen. All tissues were stored in −80 °C until analysis. Fasted blood samples were collected at 7 and 10 weeks of age for measurement of glucose, insulin and leptin according to our previous work [26].

### 2.3. Insulin Tolerance Test (ITT)

An insulin tolerance test was performed in week 9 of life (*n* = 10 rats/group). After a 7-h fast, rats received an insulin load (0.75 U/kg) through intraperitoneal injection. Blood glucose levels during the ITT were measured at 0 (baseline), 15, 30, 60, 90 and 120 min using a OneTouch^®^ Verio Blood Glucose Meter (LifeScan, Malvern, PA, USA). Insulin resistance was assessed using Homeostatic Model Assessment of Insulin Resistance (HOMA-IR) using the formula: (fasting insulin concentration × fasting glucose concentration)/22.5 as reported previously [27].

### 2.4. Food and Fluid Intake

Water and food intake were measured by weighing feed cups and water bottles throughout the study at 4 different time points (week 5 of life–during their second antibiotic pulse; week 6 of life–during the third antibiotic pulse; week 8 of life–before insulin tolerance test; and week 10 of life–end of the study).

### 2.5. Body Weight and Composition

Body weight was measured weekly throughout the study. At week 7 and week 10 of life, animals were lightly anaesthetized with isoflurane and body composition was measured via dual energy X-ray absorptiometry (DXA) scan with software for small animals (Hologic ODR 4500, Marlborough, MA, USA).

### 2.6. Serum LPS

Blood was collected via tail bleed at week 7 and portal vein at week 10 of life. Blood was centrifuged at 1200× *g* for 10 min and serum stored at −80 °C until analysis. For the analysis, samples were heated for 1 h at 70 °C and LPS measured using PyroGene Recombinant Factor C Endotoxin Detection Assay (Lonza, Basel, Switzerland) as described previously [16,28].

### 2.7. Real-Time PCR Analysis

Hypothalamic, ileal and liver samples were processed for real-time PCR as previously described [29]. Briefly, total RNA was extracted using TRIzol reagent (Invitrogen, Carlsbad, CA, USA) and reverse transcription to cDNA was performed using 2 μg of total RNA and cDNA synthesis kit (Invitrogen). Primers for liver-related genes (*IRS-1* and *IRS-2* and housekeeping: *GAPDH*), ileal genes (*ZO-1*, *occludin*, *Muc2* and *MMP9* and housekeeping: *18S*) and hypothalamus-related genes (*POMC*, *AGRP*, *NPY*, *IL-10*, *IRS-1*, *IRS-2* and housekeeping: *β-actin*) are listed in Supplemental Table S1. The mRNA levels were calculated using the 2^−ΔCT^ method [16].

### 2.8. Fecal Collection and 16S rRNA Illumina Sequencing

Fecal samples were collected repeatedly throughout the study; after the first (day 22 of life), second (day 31 of life), and third (day 40 of life) antibiotic pulse and at the end of study (beginning of week 10/day 64 of life). Using ~250 mg of fecal matter, total bacterial DNA was extracted using a FastDNA Spin Kit for feces with bead beating (MP Biomedicals, Solon, OH, USA). Fecal DNA was quantified (PicoGreen kit, Invitrogen, Carlsbad, CA, USA) and diluted to 20 ng/μL for sequencing. Microbial sequencing was performed on the MiSeq Illumina platform at the Centre for Health Genomics and Informatics (University of Calgary). The V3 and V4 regions of the 16S rRNA gene were amplified and the protocol involved a two-step, tailed PCR approach that generated ready-to-pool amplicon libraries as described previously [17]. The pooled and indexed library set was denatured, diluted, and sequenced in paired-end modus on an Illumina MiSeq (Illumina Inc., San Diego, CA, USA). Sequences were checked for quality, trimmed, merged, and checked for chimeras using the DADA2 version 1.12 [30] and phyloseq [31] packages for R (R Development Core Team; http://www.R-project.org). A bacterial community matrix was built from the resulting unique Amplicon Sequence Variants (ASV). To reduce biases introduced by DNA amplification (i.e., PCR) and sequencing errors, we excluded any ASV that was found less than five times in the community matrix. This resulted in a final dataset of 4,336,788 quality sequences and 2280 ASVs. The number of sequences per sample varied from 2732 to 20,533, with a mean of 12,945.64.

### 2.9. Statistical Analysis—Biological and qPCR Outcomes

All data is presented as mean ± standard error of the mean (SEM). Boxplots were made to identify outliers and normality was assessed using the Shapiro-Wilk test. If the data was normally distributed (*p* > 0.05), parametric one-way analysis of variance (ANOVA) with Tukey’s post-hoc tests was used. For longitudinal and timed data (body weights, ITT), statistical tests on univariate response variable were performed using a linear mixed-model for repeated measures, followed by an ANOVA with Tukey’s post hoc when appropriate. The lean reference group was not included into statistical analysis. In all tests, significance was set at *p* < 0.05. Statistical analyses and graphs were made using Prism version 7.0d (GraphPad Software, La Jolla, CA, USA).

### 2.10. Statistical Analysis (16S rRNA Illumina Sequencing)

To account for potential heteroskedasticity in community beta-diversity dispersion and avoid the loss of information through rarefaction [32], we performed a variance stabilizing transformation [32,33] prior to any statistical tests. Changes in gut bacterial community structure (beta-diversity) were assessed statistically using Permutational Multivariate ANalysis Of Variance (PERMANOVA) and visualized using Principal Coordinates Analysis (PCoA) based on Bray-Curtis dissimilarities. To explore further the changes in taxonomical community structure, we tested for significant changes in relative abundance of the 15 most dominant bacterial families using non-parametric Kruskal-Wallis tests followed by post-hoc Dunn tests with Benjamin-Holmes False Discovery Rate (FDR) correction. To estimate gut bacterial alpha-diversity, we measured the Chao1 (richness) and Shannon indices. We used an ANOVA on a linear mixed-model to test for significant differences in alpha-diversity between treatments and time points, followed by a Tukey’s post-hoc test. Subject ID was implemented as a random factor to account for repeated measures in our dataset. All data is presented as mean ± standard error of the mean (SEM). The data set is available at NCBI SRA accession: PRJNA641149.

## 3. Results

### 3.1. Pulsed Early Life Antibiotic Exposure Increases Body Weight and Leads to Insulin Resistance

**Figure 1:**
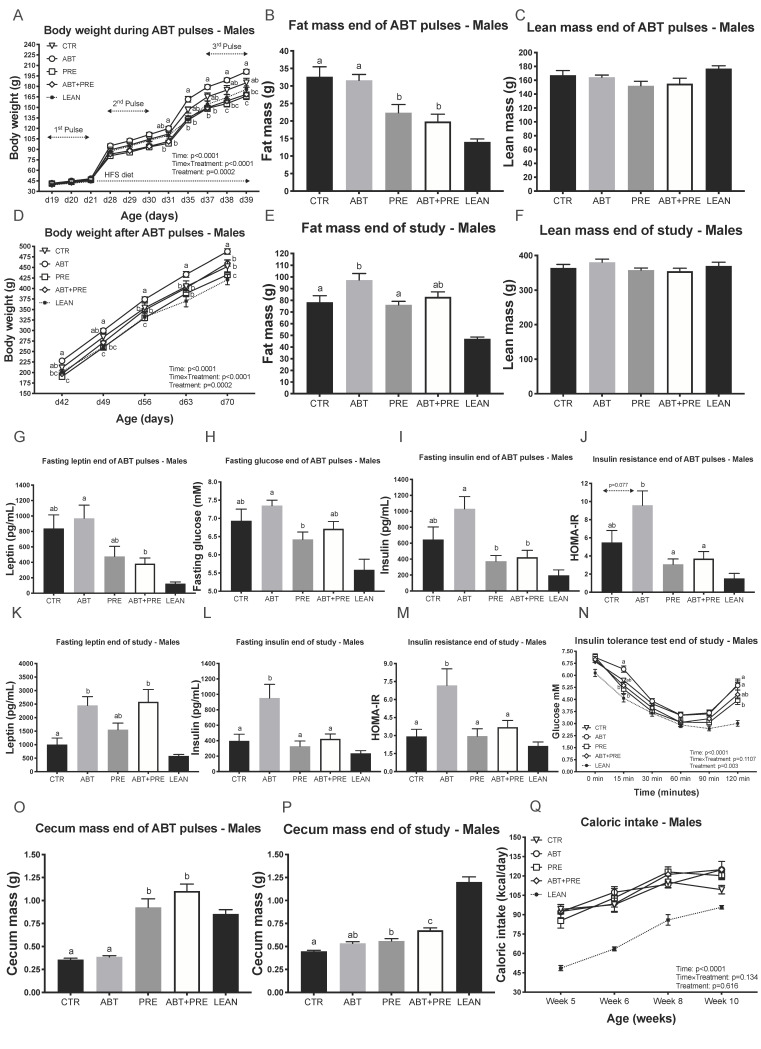
Pulsed early life antibiotic exposure and weaning onto a high fat/sucrose diet increases body weight, fat mass and insulin levels/resistance in males, all reversible with prebiotic co-administration. (**A**) Body weight of males during antibiotic pulses (*n* = 8 rats/group). (**B**) Fat mass and (**C**) Lean mass of males at the end of antibiotic pulses (*n* = 7–10 rats/group). (**D**) Body weight of males after antibiotic pulses (*n* = 8 rats/group). (**E**) Fat mass and (**F**) Lean mass of males at the end of the study (*n* = 7–10 rats/group). Portal vein leptin (**G**) and insulin (**I**) levels of males measured at the end of antibiotic pulses (*n* = 7–10 rats/group). (**H**) Fasting glucose of males measured at the end of antibiotic pulses via tail bleed (*n* = 8–10 rats/group). (**J**) Insulin resistance of males at the end of antibiotic pulses (*n* = 8–9 rats/group). Portal vein leptin (**K**) and insulin (**L**) levels of males at the end of the study (*n* = 7–9 rats/group). (**M**) Insulin resistance of males at the end of the study (*n* = 8 rats/group). (**N**) Glucose response to insulin in males measured by ITT at the end of the study (*n* = 8–10 rats/group). (**O**,**P**) Cecum mass in males at the end of antibiotic pulses (**O**) and end of study (**P**) (*n* = 7–10 rats/group). (**Q**) Average caloric intake (kilocalories) of males calculated as the average of energy intake over 4 days measured at 4 different weeks of life (*n* = 6–8 rats/group). Results are shown as mean ± SEM. The superscripts ^a,b^ indicate significant differences between groups where labelled means without a common superscript letter differ, *p* < 0.05 (i.e., ‘a’ and ‘b’ differ; ‘ab’ does not differ from ‘a’ or ‘b’). CTR, control; ATB, antibiotic; PRE, prebiotic; ATB + PRE, antibiotic + prebiotic; LEAN, lean control; d, day of life.

Previous animal studies demonstrated increased body weight after sub-therapeutic antibiotic exposure [12,34], therefore we aimed to investigate whether therapeutic doses of azithromycin resulted in a similar phenotype. Male rats given antibiotics alone (ABT group) were ~20% heavier than the prebiotic groups (PRE, ABT + PRE) by the end of the second antibiotic pulse (d31, Figure 1A) and remained heavier until the end of the pulses (Figure 1A). The differences in body weight between ABT and prebiotic groups were due to increased fat mass (Figure 1B) and not lean mass (Figure 1C). In addition, ABT + PRE had lower leptin levels (Figure 1G), PRE had lower fasting glucose (Figure 1H), and both prebiotic groups had lower fasting insulin (Figure 1I) and were more insulin sensitive (Figure 1J) compared to ABT at the end of antibiotic pulses. Once the antibiotics/prebiotics were discontinued and the experimental groups continued on the HFS diet, the ABT group became heavier than all other groups (Figure 1D) despite no differences in caloric intake between the groups (Figure 1Q). To determine if higher body weight in ABT rats was due to accelerated weight gain over time or a reflection of early weight changes, we assessed weight gain between day 42 and day 70. There were no significant differences between ABT (287 ± 4 g), PRE (267 ± 7 g), ABT + PRE (292 ± 7 g) and CTR (267 ± 11 g), however, ABT and ABT + PRE did have significantly greater weight gain compared to Lean Control (245 ± 10 g; *p* < 0.007) whereas the difference between PRE, CTR and Lean Control did not differ (*p* > 0.3). The increase in body weight in the ABT group was due to increased fat mass (Figure 1E) and not lean mass (Figure 1F). Interestingly, the male antibiotic groups (ABT, ABT + PRE) had increased leptin levels compared to CTR at the end of the study (Figure 1K), however, fasting insulin levels (Figure 1L) as well as insulin resistance (Figure 1M) was strictly increased in the ABT group. During the insulin tolerance test, glycemia was lower in PRE and ABT + PRE at 15 min and lower in PRE compared to ABT at 120 min following the insulin load indicating reduce insulin sensitivity (Figure 1N). Higher serum LPS levels were seen in ABT group at week 10 compared to CTR and PRE groups (Supplemental Figure S1A), but not at week 7 (Supplemental Figure S1B). While the animals were on a prebiotic diet, cecum mass was increased in the prebiotic groups (Figure 1O) and remained significantly heavier in ABT + PRE compared to ABT and CTR until the end of study; PRE was heavier than CTR (Figure 1P).

**Figure 2:**
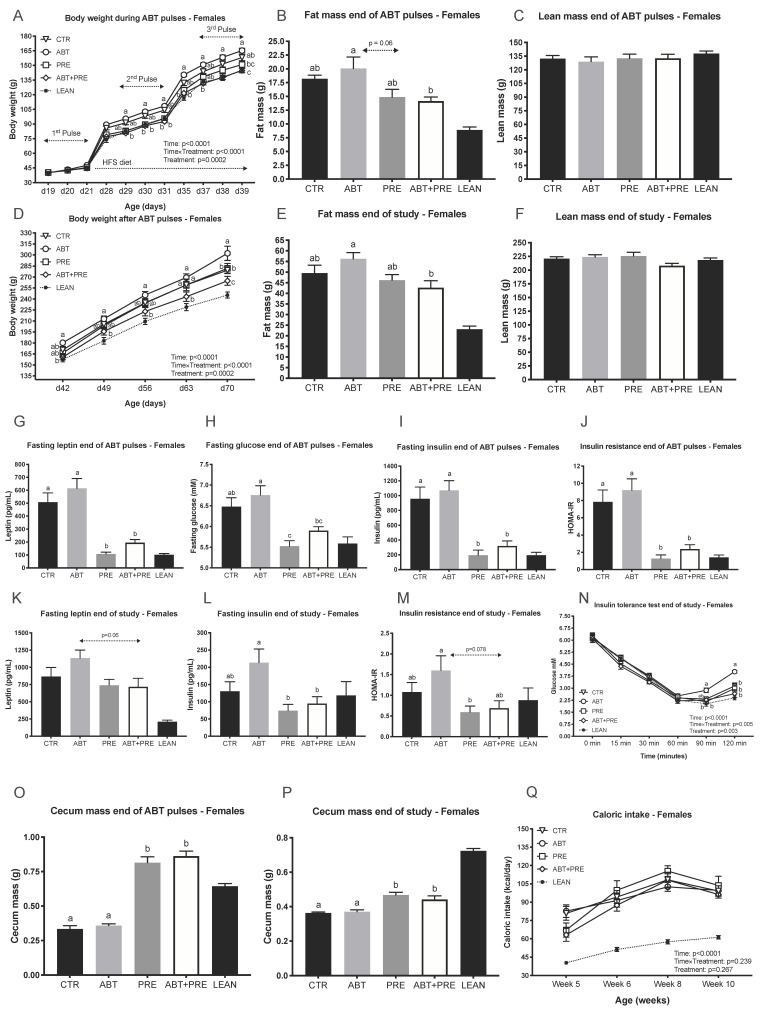
Pulsed early life antibiotic exposure and weaning onto a high fat/sucrose diet increases body weight and insulin levels/resistance in females, all reversible with prebiotic co-administration. (**A**) Body weight of females during antibiotic pulses (*n* = 9 rats/group). (**B**) Fat mass and (**C**) Lean mass of females at the end of antibiotic pulses (*n* = 7–10 rats/group). (**D**) Body weight of females after antibiotic pulses (*n* = 9 rats/group). (**E**) Fat mass and (**F**) Lean mass of females at the end of the study (*n* = 8–10 rats/group). Portal vein leptin (**G**) and insulin (**I**) levels of females measured at the end of antibiotic pulses (*n* = 8–10 rats/group). (**H**) Fasting glucose of females measured at the end of antibiotic pulses via tail bleed (*n* = 8–9 rats/group). (**J**) Insulin resistance of females was calculated at the end of antibiotic pulses (*n* = 8–10 rats/group). Portal vein leptin (**K**) and insulin (**L**) levels of females measured at the end of the study (*n* = 7–9 rats/group). (**M**) Insulin resistance of females was calculated end of study (*n* = 8–10 rats/group). (**N**) Glucose response to insulin in females measured by ITT at the end of the study (*n* = 7–10 rats/group). (**O**,**P**) Cecum mass in females at the end of antibiotic pulses (**O**) and end of study (**P**) (*n* = 8–10 rats/group). (**Q**) Average caloric intake (kilocalories) of females calculated as the average of energy intake over 4 days measured at 4 different weeks of life (*n* = 6–8 rats/group). Results are shown as mean ± SEM. The superscripts ^a,b^ indicate significant differences between groups where labelled means without a common superscript letter differ, *p* < 0.05 (i.e., ‘a’ and ‘b’ differ; ‘ab’ does not differ from ‘a’ or ‘b’). CTR, control; ATB, antibiotic; PRE, prebiotic; ATB + PRE, antibiotic + prebiotic; LEAN, lean control; d, day of life.

Similar to males, the female ABT group became ~15% heavier during the second ABT pulse (d30, Figure 2A) and remained heavier until the end of the ABT pulses compared to the prebiotic groups (PRE, ABT + PRE). Weight gain between day 42 and day 70 did not differ between ABT (137 ± 9 g), PRE (131 ± 5 g), ABT + PRE (120 ± 4 g) and CTR (121 ± 3 g), however, ABT and PRE did have significantly greater weight gain compared to Lean Control (99.9 ± 2 g; *p* < 0.002) whereas the difference between Lean Control and ABT + PRE (*p* = 0.076), CTR (*p* = 0.055) did not differ. The difference in body weight was due to lower fat mass in the prebiotic groups (Figure 2B) and not lean mass (Figure 2C). In addition, the prebiotic groups displayed lower leptin levels (Figure 2G), fasting glucose (Figure 2H), fasting insulin (Figure 2I) and insulin resistance (Figure 2J) compared to the ABT group. The metabolic profiles of prebiotic groups at the end of the ABT pulses (Figure 2G–J) matched lean control levels even though they had consumed a HFS diet for 18 days already. At the end of the study, the ABT group was heavier compared to all other groups (Figure 2D) despite no differences in their caloric intake (Figure 2Q). Fat mass was significantly lower in ABT + PRE compared to the ABT group at the end of the study (Figure 2E) with no difference in lean mass (Figure 2F). A trend (*p* = 0.06) towards a decrease in leptin was seen in the female ABT + PRE group compared to ABT group (Figure 2K). Fasting insulin was significantly reduced in both prebiotic groups compared to ABT at the end of the study (Figure 2L). HOMA-IR was also reduced in the prebiotic groups compared to ABT except that ABT + PRE was only a trend (*p* = 0.078) (Figure 2M). At the end of the ITT test, reduced insulin sensitivity was seen in the ABT group compared to all other groups (Figure 2N). No differences in serum LPS levels were detected in females (Supplemental Figure S1C,D). Prebiotic diet increased cecum weight in the prebiotic groups at the end of the pulses compared to ABT and CTR (Figure 2O) and cecum mass remained heavier at the end of the study (Figure 2P).

### 3.2. Early Life Pulsed Antibiotic Exposure Impacts Hepatic, Ileal and Hypothalamic Gene Expression

Insulin resistance was the strongest ABT-linked metabolic phenotype and we therefore explored potential mechanisms that could account for this outcome. Hepatic and hypothalamic gene expression was examined at two time points (end of antibiotic pulses and end of study) whereas ileal gene expression was investigated at the end of study when the insulin resistance phenotype was the strongest.

**Figure 3:**
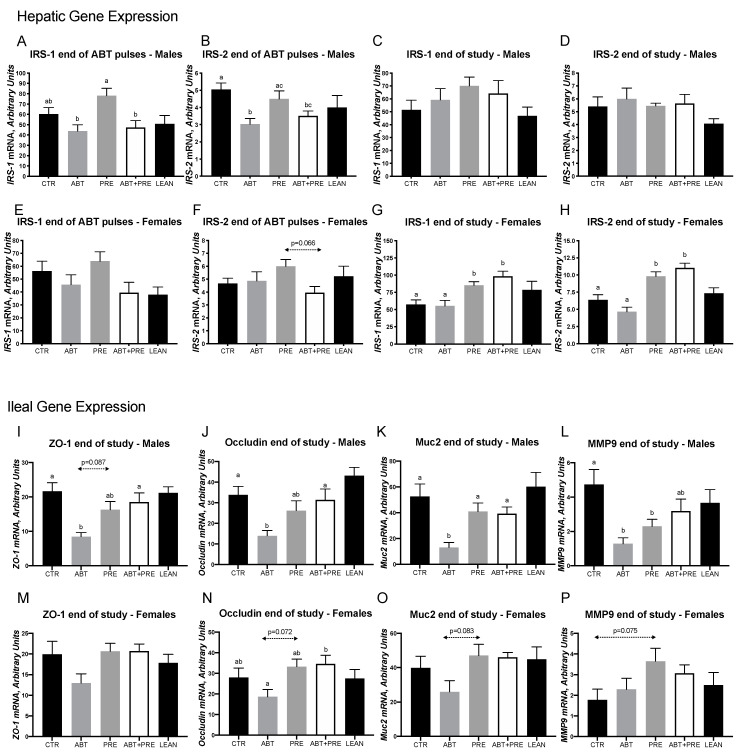
Early life pulsed antibiotic exposure impacts hepatic and ileal gene expression in males and females. Hepatic *IRS-1* (**A**) and *IRS-2* (**B**) expression in males after antibiotic pulses and at the end of the study (**C**,**D** respectively). Hepatic *IRS-1* (**E**) and *IRS-2* (**F**) expression in females after antibiotic pulses and at the end of the study (**G**,**H** respectively). Ileal *ZO-1* (**I**), *occludin* (**J**), *Muc2* (**K**) and *MMP9* (**L**) expression in males at the end of the study. Ileal *ZO-1* (**M**), *occludin* (**N**), *Muc2* (**O**) and *MMP9* (**P**) expression in females at the end of the study. Results are shown as mean ± SEM (*n* = 7–10 rats/group). The superscripts ^a,b^ indicate significant differences between groups where labelled means without a common superscript letter differ, *p* < 0.05 (i.e., ‘a’ and ‘b’ differ; ‘ab’ does not differ from ‘a’ or ‘b’). CTR, control; ATB, antibiotic; PRE, prebiotic; ATB + PRE, antibiotic + prebiotic; LEAN, lean control; *IRS-1*, insulin receptor substrate-1; *IRS-2*, insulin receptor substrate-2; *ZO-1*, zonula occludens-1; *Muc2*, Mucin 2; *MMP9*, matrix metalloproteinase 9.

In males, lower hepatic insulin receptor substrate-1 (*IRS-1*) mRNA levels were seen in the ABT group at the end of the ABT pulses compared to the PRE but PRE co-administration could not correct the lower levels as ABT + PRE was also significantly lower than PRE (Figure 3A). *IRS-2* mRNA levels were lower in ABT compared to CTR and PRE with ABT + PRE being significantly lower than CTR but not differing from PRE (Figure 3B). Differences in *IRS-1* and *IRS-2* mRNA levels were no longer present at the end of the study (Figure 3C,D). In females, *IRS-1* and *IRS-2* mRNA levels did not differ following the antibiotic pulses (Figure 3E,F) but were significantly higher in both prebiotic groups at the end of the study compared to ABT and CTR (Figure 3G,H) indicating a correction of low *IRS-1* and *IRS-2* in the ABT + PRE group.

Given our finding of greater serum LPS and metabolic endotoxemia in the ABT group, we investigated genes involved in the intestinal barrier. In males, lower mRNA levels of *ZO-1*, *occludin*, *Muc2* and *MMP9* were seen in the ABT group compared to CTR (Figure 3I–L, respectively). Prebiotic co-administration (ABT + PRE) in males normalized the ileal mRNA levels of *ZO-1*, *occlucin* and *Muc2* to CTR levels (Figure 3I–L). In females, even though mRNA levels of *ZO-1*, *occludin* and *Muc2* were the lowest in ABT, these were not significantly so (Figure 3M–O, respectively). Only for *occludin* mRNA levels was ABT + PRE significantly higher than ABT. No significant differences in *MMP9* expression were seen in females (Figure 3P).

**Figure 4:**
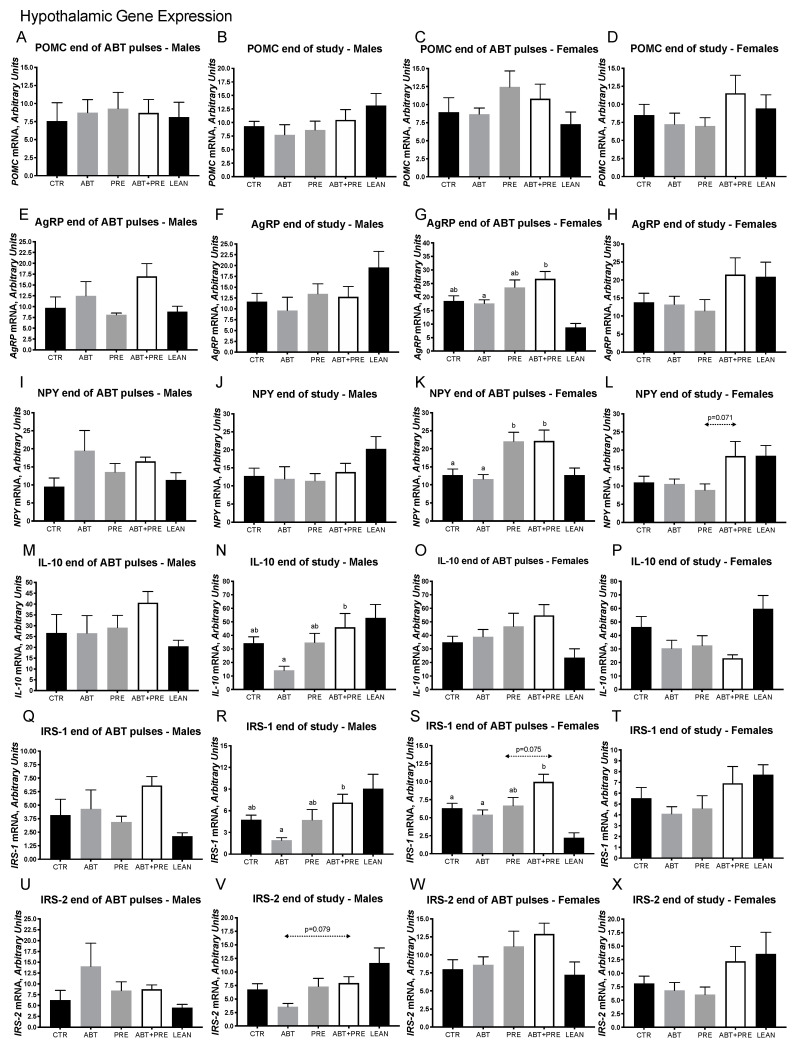
Early life pulsed antibiotic exposure impacts hypothalamic gene expression in males and females. Mean ± SE. Hypothalamic *POMC* expression in males (**A**,**B**) and females (**C**,**D**) after antibiotic pulses and at the and end of the study, respectively. Hypothalamic *AGRP* expression in males (**E**,**F**) and females (**G**,**H**) after antibiotic pulses and at the end of the study, respectively. Hypothalamic *NPY* expression in males (**I**,**J**) and females (**K**,**L**) after antibiotic pulses and at the end of the study, respectively. Hypothalamic *IL-10* expression in males (**M**,**N**) and females (**O**,**P**) after antibiotic pulses and at the end of the study, respectively. Hypothalamic *IRS-1* expression in males (**Q**,**R**) and females (**S**,**T**) after antibiotic pulses and at the end of the study, respectively. Hypothalamic *IRS-2* expression in males (**U**,**V**) and females (**W**,**X**) after antibiotic pulses and at the end of the study, respectively. Results are shown as mean ± SEM (*n* = 7–10 rats/group). The superscripts ^a,b^ indicate significant differences between groups where labelled means without a common superscript letter differ, *p* < 0.05 (i.e., ‘a’ and ‘b’ differ; ‘ab’ does not differ from ‘a’ or ‘b’). CTR, control; ATB, antibiotic; PRE, prebiotic; ATB + PRE, antibiotic + prebiotic; LEAN, lean control. *POMC*, proopiomelanocortin; *AGRP*, agouti-related peptide; *NPY*, neuropeptide Y; *IL-10*, interleukin-10; *IRS-1*, insulin receptor substrate-1; *IRS-2*, insulin receptor substrate-2.

Given the importance of the hypothalamus in maintaining energy and glucose homeostasis and the link between microbiota and central inflammation, we next investigated the expression of relevant genes in the hypothalamus (Figure 4A–T). While no differences in the expression of pro-opiomelanocortin (*POMC*) (Figure 4A,B), agouti-related peptide (*AGRP*) (Figure 4E,F) and neuropeptide Y (*NPY*) (Figure 4I,J) were seen in males at any time point, female ABT + PRE had increased expression of *AGRP* compared to ABT at the end of the ABT pulses (Figure 4G) and *NPY* expression was significantly upregulated in PRE and ABT + PRE compared to ABT at the end of the pulses (Figure 4K). Lower expression of the anti-inflammatory cytokine interleukin-10 (*IL-10*) (Figure 4N), *IRS-1* (Figure 4R) and *IRS-2* (*p* = 0.079; Figure 4V) was seen in ABT males compared to ABT + PRE males at the end of the study. Similarly, lower hypothalamic expression of *IRS-1* (Figure 4S) was seen in the female ABT group at the end of the ABT pulses compared to the ABT + PRE group but not at the end of the study (Figure 4T). No differences were seen in *POMC* (Figure 4C,D), *IL-10* (Figure 4O,P) and *IRS-2* (Figure 4W,X) expression in females at any time point. Likewise, no differences were seen at the end of the ABT pulses for *IL-10* (Figure 4M), *IRS-1* (Figure 4Q) and *IRS-2* (Figure 4U) expression in males.

### 3.3. Antibiotic and/or Prebiotic Treatment and Not Time Was the Main Driver of Gut Bacterial Community Structure in Males and Females

**Figure 5:**
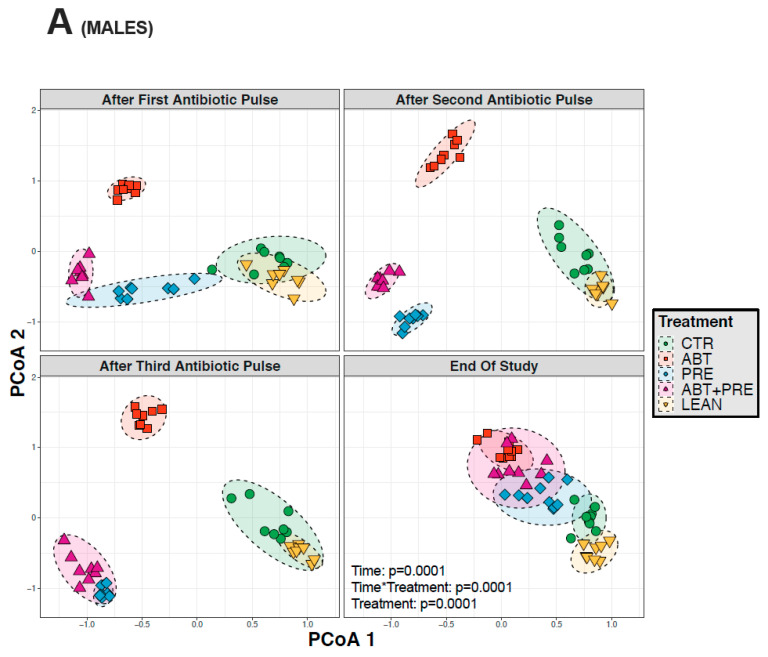

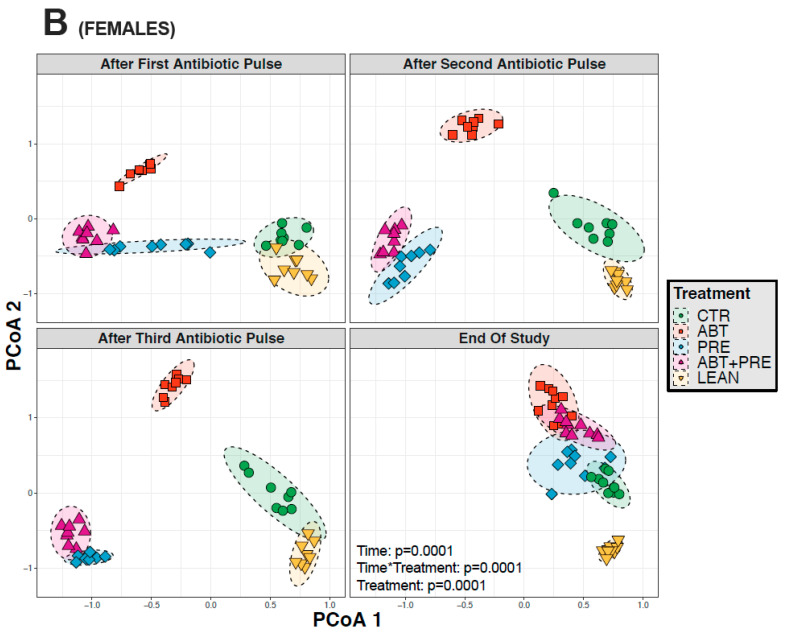
Between-group variations in beta-diversity of gut bacterial communities in male and female rats at different time points: after the first, second, third antibiotic exposure, and at the end of the study. (**A**) Principal coordinates analysis (PCoA) ordination of variation in beta-diversity of gut bacterial communities based on Bray-Curtis dissimilarities among male (**A**) and female (**B**) rats (*n* = 8–10 rats per group/time point). Statistical significance of the effect of treatment and experimental time points on gut bacterial community structure was tested with a Permutational Analysis of Variance (PERMANOVA). CTR, control; ABT, antibiotic; PRE, prebiotic; ABT + PRE, antibiotic + prebiotic; LEAN, lean control.

**Figure 6:**
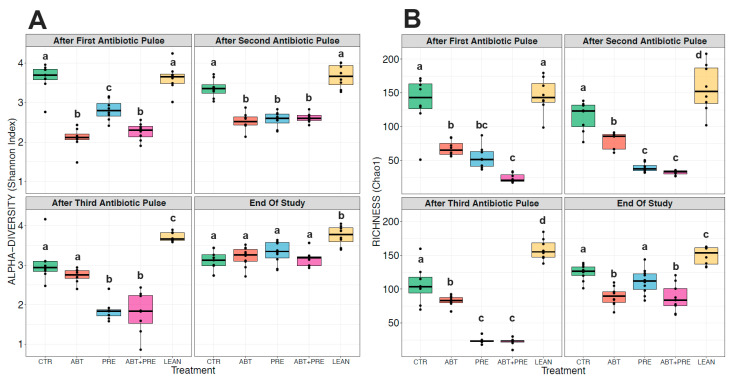

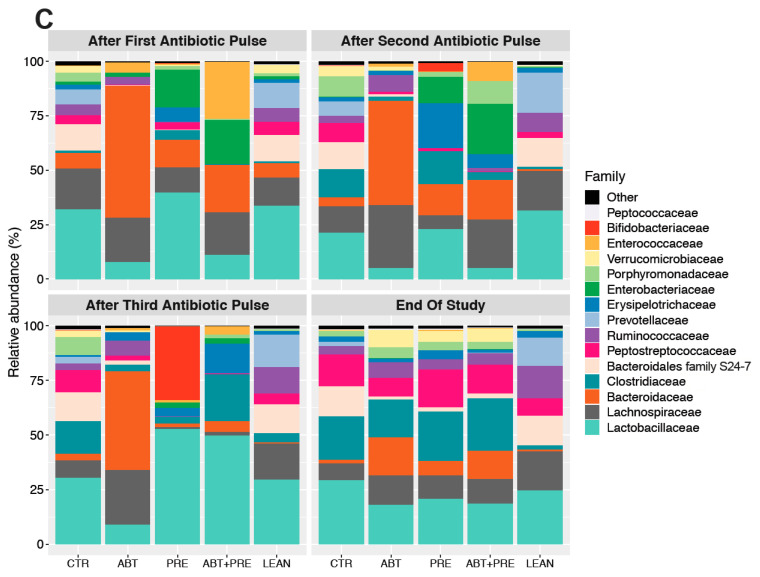
Between-group variations in alpha-diversity of gut bacterial communities and relative abundances of the 15 most abundant bacterial families in male rats over time. Shannon diversity (**A**) and Chao1 estimated richness (**B**) display between-group differences in alpha-diversity in male rats over time (*n* = 8–10 rats per group/time point). ANOVA with Tukey post-hoc test; *p* < 0.05. (**C**) Between-group relative abundances of the 15 most abundant bacterial families in male rats after the first, second, third antibiotic exposure and at the end of the study. Kruskal-Wallis test with Dunn post-hoc tests and Benjamin-Holmes False Discovery Rate (FDR) correction; *p* < 0.05. The superscripts ^a,b,c,d^ indicate significant differences between groups where labelled means without a common superscript letter differ, *p* < 0.05 (i.e., ‘a’ and ‘b’ differ; ‘ab’ does not differ from ‘a’ or ‘b’) (To see significant differences between groups: Supplemental Table S2). CTR, control; ABT, antibiotic; PRE, prebiotic; ABT + PRE, antibiotic + prebiotic; LEAN, lean control.

**Figure 7:**
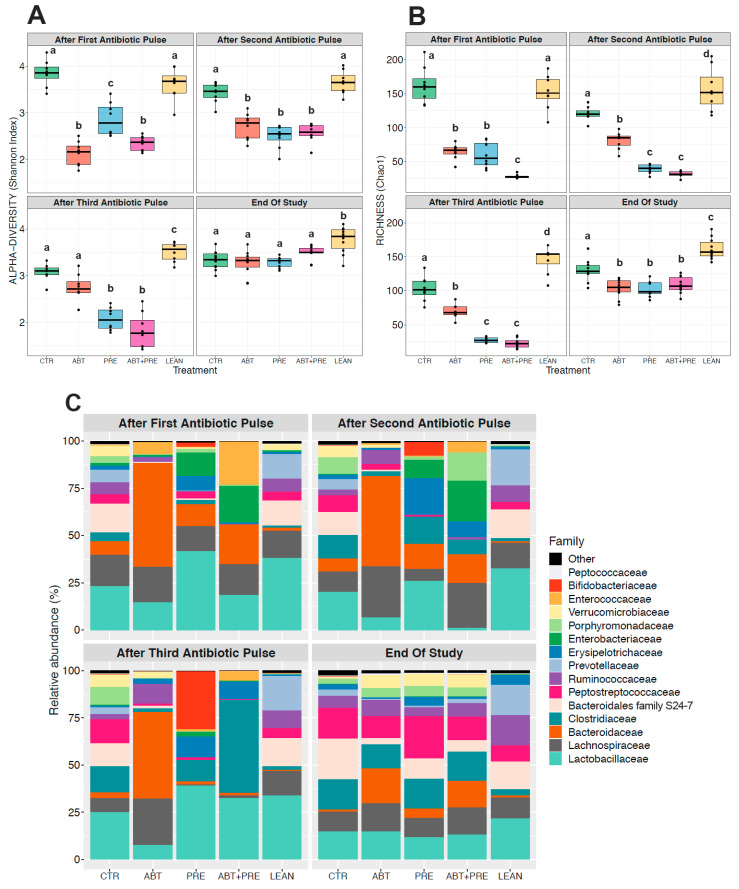
Between-group variations in alpha-diversity of gut bacterial communities and relative abundances of the 15 most abundant bacterial families in female rats over time. Shannon diversity (**A**) and Chao1 estimated richness (**B**) display between-group differences in alpha-diversity in female rats over time (*n* = 8–10 rats per group/time point). ANOVA with Tukey post-hoc test; *p* < 0.05. (**C**) Between-group relative abundances of the 15 most abundant bacterial families in female rats after the first, second, third antibiotic exposure and at the end of the study. Kruskal-Wallis test with Dunn post-hoc tests and Benjamin-Holmes False Discovery Rate (FDR) correction; The superscripts ^a,b,c,d^ indicate significant differences between groups where labelled means without a common superscript letter differ, *p* < 0.05 (i.e., ‘a’ and ‘b’ differ; ‘ab’ does not differ from ‘a’ or ‘b’). (To see significant differences between groups: Supplemental Table S3). CTR, control; ABT, antibiotic; PRE, prebiotic; ABT + PRE, antibiotic + prebiotic; LEAN, lean control.

Many environmental and lifestyle factors such as diet and antibiotics can profoundly alter gut microbiota throughout life [35]. We therefore collected fecal samples after each PAT and monitored changes over time. PERMANOVA analysis showed that treatment was the strongest driver of bacterial community structure explaining 35.5% in males and 33.8% in females (β diversity, *p* = 0.001), whereas the experimental time point explained 12.2% of the variation in males (*p* = 0.001; Figure 5A) and 13.2% in females (*p* = 0.001; Figure 5B). The response in community structure in males and females changed over time depending on the treatment (R^2^ = 18.3% for males; R^2^ = 19.4%, for females; *p* = 0.001) and the model explained a total of 66% (males) and 66.4% (females) of the changes in the bacterial community assembly. A reduction in alpha-diversity in males (Figure 6A,B) and females (Figure 7A,B) was seen in antibiotic and prebiotic groups when compared to controls, however, differences between groups disappeared once antibiotics and/or prebiotics were discontinued at the end of the study.

To further explore the variation in gut bacterial composition, the relative abundance of the 15 most dominant bacterial families was analyzed per time point and per treatment in males (Figure 6C) and females (Figure 7C). While control groups (CTR and LEAN) and the prebiotic alone group (PRE) were defined by higher levels of *Lactobacillaceae* after the antibiotic pulses in males (Figure 6C; Supplemental Table S2) and females (Figure 7C, Table S3), the antibiotic group (ABT) was dominated by *Bacteroidaceae* in both sexes. Co-administration of prebiotics with antibiotics (ABT + PRE) rescued low *Lactobacillaceae* levels and reduced high *Bacteroidaceae* levels, but only after the third antibiotic pulse in males (Figure 6C; Supplemental Table S2) and females (Figure 7C, Supplemental Table S3). By the third antibiotic pulse, prebiotic co-administration also corrected the bloom in *Lachnospriaceae* and reduction in *Clostridiaceae*_1 observed in the ABT group in males and females (Supplemental Tables S2 and S3). Prebiotics (ABT + PRE group) were not able to increase levels of *Porphyromonadaceae*, *Prevotellaceae*, and *Peptostreptococcaceae* which were depleted in ABT males (Figure 6C; Supplemental Table S2) and females (Figure 7C; Supplemental Table S3) after the third antibiotic pulse. Once the antibiotic treatment and prebiotic supplementation were discontinued and fecal samples were analyzed at the end of the study, most of the bacterial differences between groups disappeared in males and females, however, higher *Bacteroidaceae* levels persisted in antibiotic groups (ABT and ABT + PRE) compared to others.

### 3.4. Treatment Disrupts Gut Bacterial Community Maturation in Males and Females

**Figure 8:**
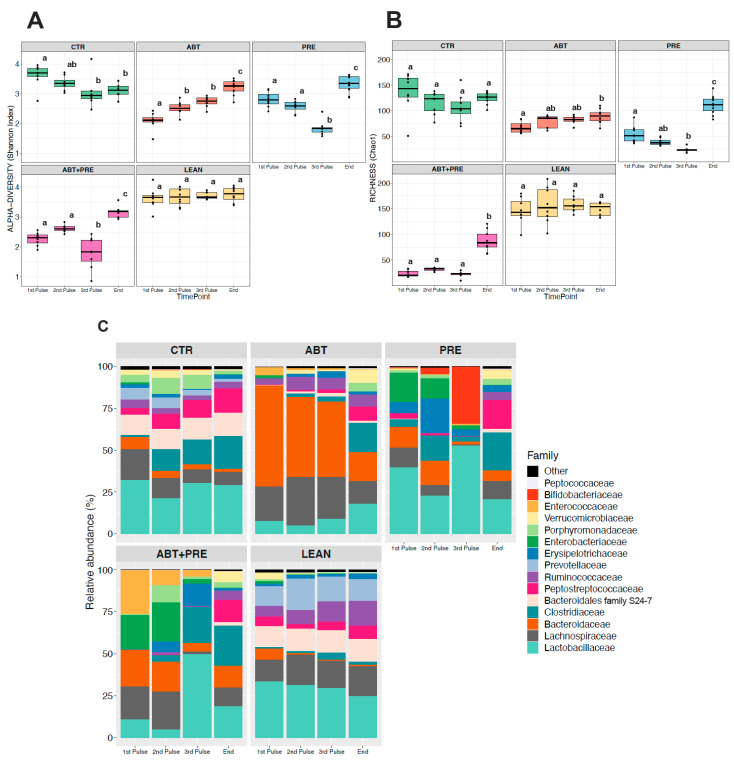
Within group variations in alpha-diversity of gut bacterial communities and relative abundances of the 15 most abundant bacterial families in male rats over time. Shannon diversity (**A**) and Chao1 estimated richness (**B**) display within group differences in alpha-diversity in male rats over time (*n* = 8–10 rats per group/time point). ANOVA with Tukey post-hoc test; *p* < 0.05. (**C**) Within group relative abundances of the 15 most abundant bacterial families in male rats over time. Kruskal-Wallis test with Dunn post-hoc tests and Benjamin-Holmes False Discovery Rate (FDR) correction; the superscripts ^a,b,c^ indicate significant differences between time points where labelled means without a common superscript letter differ, *p* < 0.05 (i.e., ‘a’ and ‘b’ differ; ‘ab’ does not differ from ‘a’ or ‘b’). CTR, control; ABT, antibiotic; PRE, prebiotic; ABT + PRE, antibiotic + prebiotic; LEAN, lean control. 1st Pulse, After first antibiotic pulse; 2nd Pulse, After second antibiotic pulse; 3rd Pulse, After third antibiotic pulse; End, End of Study.

**Figure 9:**
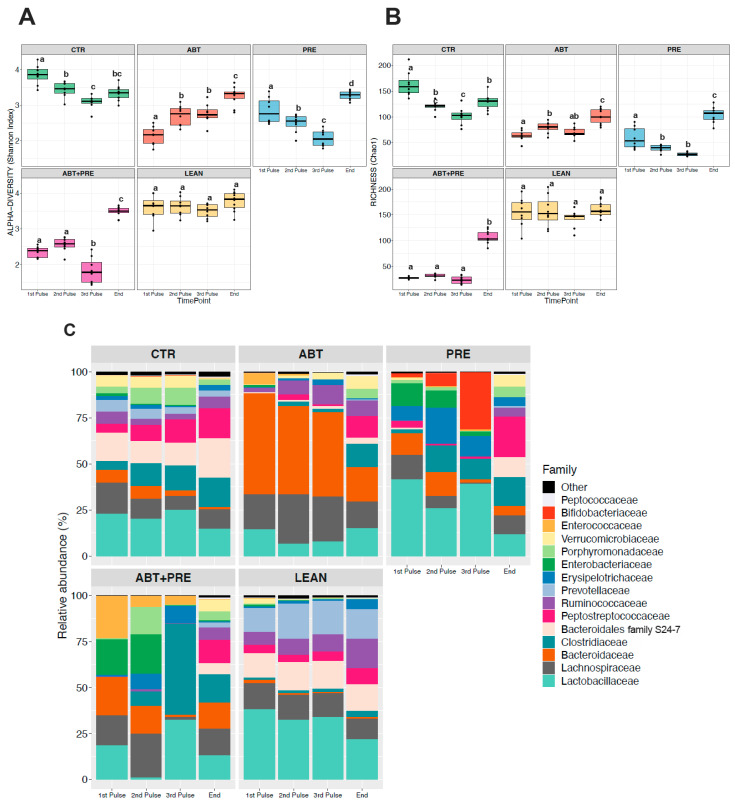
Within group variations in alpha-diversity of gut bacterial communities and relative abundances of the 15 most abundant bacterial families in female rats over time. Shannon diversity (**A**) and Chao1 estimated richness (**B**) display within group differences in alpha-diversity in female rats over time (*n* = 8–10 rats per group/time point). ANOVA with Tukey post-hoc test; *p* < 0.05. (**C**) Within group relative abundances of the 15 most abundant bacterial families in female rats over time. Kruskal-Wallis test with Dunn post-hoc tests and Benjamin-Holmes False Discovery Rate (FDR) correction; the superscripts ^a,b,c,d^ indicate significant differences between time points where labelled means without a common superscript letter differ, *p* < 0.05 (i.e., ‘a’ and ‘b’ differ; ‘ab’ does not differ from ‘a’ or ‘b’). CTR, control; ABT, antibiotic; PRE, prebiotic; ABT + PRE, antibiotic + prebiotic; LEAN, lean control. 1st Pulse, After first antibiotic pulse; 2nd Pulse, After second antibiotic pulse; 3rd Pulse, After third antibiotic pulse; End, End of Study.

Considering microbial communities that reside in the gut, environmental perturbations (i.e., antibiotics) and lack of microbiota stability in the first three years of life [36], we decided to investigate microbial maturation/development for each experimental group separately over time (Figure 8 and Figure 9). Antibiotic male (Figure 8C) and female (Figure 9C) gut microbiota development was disrupted with antibiotics, demonstrated by the bloom in *Bacteroidaceae* after each antibiotic pulse that contrasted with the control groups (CTR and LEAN) where levels remained relatively stable over time. Furthermore, *Lactobacillaceae* levels decreased over time in control/lean control groups in males (Figure 8C) and females (Figure 9C), peaking immediately after weaning (1st pulse). However, in antibiotic groups, the trend was the opposite as the highest levels of *Lactobacillaceae* were seen at the end of study, especially in males (Figure 8C). *Bacteroidales* family *S24-7* and *Peptostreptococcaceae* were depleted in antibiotic/prebiotic groups during the intervention (1st–3rd pulse) in males (Figure 8C) and females (Figure 9C) and levels increased only at the end of the study. On the contrary, control/lean control groups had higher levels of *Bacteroidales* family *S24-7* and *Peptostreptococcaceae* and these taxa were stable over time (1st pulse–end of the study). Prebiotic group (PRE) gut microbiota development also differed from control and antibiotic groups (CTR/LEAN/ABT) with higher *Bifidobacteriaceae* levels seen after the 3rd pulse in males (Figure 8C) and females (Figure 9C). However, when prebiotics were co-administered with antibiotics (ABT + PRE), a bloom in *Clostridiaceae* was seen instead in males (Figure 8C) and females (Figure 9C). Interestingly, while a HFS diet in control (CTR) and prebiotic groups (PRE and ABT + PRE) decreased α-diversity over time (from 1st until 3rd pulse) in males (Figure 8A,B) and females (Figure 9A,B), the opposite occurred in ABT groups as α-diversity increased.

## 4. Discussion

Antibiotics are life-saving drugs, but only recently has the impact of early-life antibiotic treatment on gut microbiota development and its metabolic consequences been described [10,12]. Here we investigated a therapeutic dose of an antibiotic class commonly used in human children with the administration regimen mimicking that used in pediatric populations (i.e., use of an antibiotic with a longer half-life which enables a short duration of therapy and once per day administration) [37]. In our study, pulsed-antibiotic azithromycin treatment early in life affected host body weight, body composition, insulin resistance, expression of select genes in the liver, ileum and hypothalamus, as well as gut microbiota even after the antibiotic treatments were stopped. Although the negative effects of pulsed therapeutic-dose antibiotic treatment have been described [10], our study provides novel insight into the role of prebiotics in mitigating the adverse effects of early postnatal antibiotic treatment on weight gain and insulin resistance.

Our study demonstrates that early life pulsed antibiotic exposure (PAT) changes the capacity of the animals to respond to stressors such as a HFS diet, a finding reported previously [10]. Notably, antibiotic increased body weight in males and females following consumption of the HFS diet, with males having significantly increased fat mass compared to all other groups. While Nobel et al. [10] investigated PAT in young female mice, our study included both male and female rats and demonstrated a stronger phenotype in males after PAT than females. Our results are in line with previous studies employing sub-therapeutic antibiotic exposures in animals as well as human studies, where boys/males [12,38,39,40,41,42,43] were more prone to obesity upon early-life antibiotic exposure. These studies showed that adverse effects seen in males are rarely mirrored in females and the reasons are poorly understood. Given that females respond differently to environmental stressors such as diet/physical activity/stress [44,45], it is possible that the metabolic consequences of antibiotics are also sex-specific or that phenotypes develop later in females than males and would have been missed with our early time points. Several mechanisms have been proposed by others to explain the weight gain observed after early life antibiotic exposure [46]. Specifically, bacteria increase their energy harvesting capacity from diet, the number of health promoting bacteria decreases, metabolic signalling changes, hepatic lipogenesis increases, intestinal permeability increases and immune defense is impaired after antibiotic exposure [46].

Our study suggests the proposed mechanisms could also encompass insulin resistance and altered expression of hepatic, ileal and hypothalamic genes involved in metabolic regulation. Increased fasting insulin levels/insulin resistance were seen at the end of the antibiotic pulses and the end of the study in ABT males. It is plausible that the insulin resistance was worse in males because of the lower expression of insulin receptor substrates and ileal proteins involved in gut barrier integrity. Specifically, we saw lower hepatic *IRS-2* expression at the end of the antibiotic pulses in ABT males compared to controls, but we did not observe this trend in females. Similarly, ABT males showed a trend towards lower hypothalamic *IRS-1/2* expression, whereas females did not. In addition, lower ileal gene expression of tight junction (*ZO-1*, *occludin*) and epithelial barrier (*Muc2*, *MMP9*) proteins was observed in the male ABT group compared to control, which probably contributed to the increased gut permeability. As reviewed previously [46], another putative mechanism for increased insulin resistance seen after antibiotic exposure could be metabolic endotoxemia. The authors suggested that increased inflammation caused by translocation of LPS from Gram-negative bacteria present in the gut leads to systemic inflammation and consequently to insulin resistance [46]. It is known that pro-inflammatory cytokines such as interleukin-6 and/or tumor necrosis factor (TNF) interfere with insulin signaling [47], thereby reducing insulin sensitivity. Furthermore, high-fat/high-carbohydrate diets increase gut inflammation and permeability [48], thus enabling increased LPS translocation to the blood stream. Increased plasma LPS along with greater gut permeability leads to inflammation, weight gain, fasting hyperglycaemia and hyperinsulinemia [49]. Since male ABT animals in our study had ~10× greater fecal relative abundance of Gram-negative *Bacteroidaceae* (a source of LPS), had lower ileal expression of tight junction/gut barrier proteins and were on a HFS diet, it is possible that a leaky gut could have led to higher levels of LPS in our ABT group contributing to low-grade inflammation, increased fat mass and insulin resistance. Interestingly, LPS levels at the end of the study in males (Supplemental Figure S1B) mirrored body fat levels detected in those animals (Figure 1E).

In addition to the host physiology, PAT also had a major impact on the gut microbial ecosystem. As shown previously in female mice [10], a reduction in alpha-diversity (richness and Shannon index) was seen immediately after the first PAT, but the differences between groups were minimized by the end of study in both sexes due to the strong impact of the HFS diet on microbial composition. In addition, our beta-diversity analysis (PCoA plots) showed progressive separation of antibiotic animals from controls with each PAT, indicating that the alterations to microbial composition were driven by antibiotics and are cumulative through time. We hypothesise that low alpha-diversity in combination with altered beta-diversity is due to the extremely high abundance of *Bacteroidaceae* after PAT, separating PAT animals from other groups. Furthermore, PAT changed the typical microbiota responses to a HFS diet, a finding also reported previously [10]. The typical response to a HFS diet would be an increase in Firmicutes at the expense of Bacteroidetes with a simultaneous decrease in microbial diversity [2]. But in our PAT animals, a spike in *Bacteroidaceae* (Bacteroidetes) and depletion in *Lactobacillaceae* (Firmicutes) with a concurrent increase in alpha-diversity was seen during the PAT pulses and HFS diet consumption. One possible explanation is the mode of action of azithromycin; since it largely targets Gram-positive bacteria [50] such as Firmicutes, an opportunistic bloom of Gram-negatives (Bacteroidetes) could be expected. Depleting *Lactobacillus* in PAT animals early in life might partially explain increased weight gain at the end of study in both sexes as low levels of *Lactobacillus* present during the developmental window in another study led to increased adiposity/weight later in life [12]. Importantly, although microbial community composition largely recovered after cessation of the treatments and converged across treatment groups in adulthood, transient early changes in the microbiota appear to induce permanent metabolic alterations. The ability of a transient insult to the developing gut microbiota (e.g., antibiotics) to induce long-term effects on body composition is consistent with previous work by Cox et al. [12] showing that exposure to low-dose penicillin before weaning perturbs the microbiota in mice and is sufficient to cause long lasting metabolic changes.

A critical element of a healthy microbiota is ecosystem stability which is defined by the ability of the community to remain unchanged during perturbations (resistance) and the capacity to return to initial state after the insult (resilience) [51]. At birth, a simple gut bacterial community is established with low diversity, low bacterial load and low resilience [51]. While minor fluctuations to a certain extent are expected, major pulsed perturbations such as antibiotics or continuous perturbations (HFS diet) may result in an ill-defined state of the intestinal microbial community (dysbiosis) contributing to disease, especially early in life when the gut microbiota is still being established [51]. The so-called “insurance hypothesis” defines a strong relationship between microbial diversity and ecosystem stability meaning that communities containing many species have greater capacity to return to a stable equilibrium after the insult [51]. Furthermore, development of gut microbiota is directional indicating that the growth of certain species is dependent on the presence of other species (structured temporal succession). For example, the presence of oxygen in the intestine after birth promotes the growth of facultative anaerobic bacteria (i.e., *Lactobacillus* spp.) until the oxygen reserves are depleted and then anaerobic bacteria (i.e., Bacteroidetes) replace them [51]. In our study, the opposite was observed in antibiotic animals with high levels of anaerobic *Bacteroidaceae* and low levels of *Lactobacillaceae* demonstrating altered establishment of gut microbiota. Furthermore, *Bacteroidales* family *S24-7* and *Peptostreptococcaceae* were under-represented in ABT groups when compared to controls over time. Interestingly, similar microbial profiles as in our antibiotic-treated animals were seen in a colitis mouse model. Expansion of *Bacteroidaceae* and depletion of commensal bacteria *Bacteroidales* family *S24-7* and *Lactobacillus* species was reported in mice with colitis [52], further suggesting dysbiotic maturation of gut microbiota in our ABT animals. In line with this, it was previously reported that individuals with low bacterial richness and *Bacteroides*-dominated community had increased inflammatory markers, lower functional redundancy and lower resistance [53]. In our study, ABT males and females had *Bacteroides*-dominated intestinal communities in combination with low richness. It is therefore possible that microbial development disruptions with antibiotics early in life contributed to metabolic impairments (insulin resistance) observed in antibiotic animals. In humans, antibiotic use in the first year of life was associated with metabolic syndrome later in life [54] and unfortunately, the highest antibiotic use seen in humans occurs in the first two years of life [55].

Prebiotic co-administration in our study reduced body weight and fat mass, improved insulin sensitivity, modified gut microbiota composition and altered the expression of relevant genes in the liver, ileum and hypothalamus. A possible mechanism for these benefits is the ability of prebiotics to increase the production of GLP-2 which in turn improves intestinal barrier integrity and insulin sensitivity and reduces inflammatory markers and oxidative stress [56]. Chronic administration of GLP-2 to *ob/ob* mice decreased plasma LPS level by 50%, reduced plasma inflammatory markers and increased the gene expression of tight junction proteins (*ZO-1* and *occludin*) [56]. In another study [17], OFS supplementation improved intestinal permeability, decreased LPS levels and consequently led to lower body weight and fat mass. We confirm lower LPS levels and fat mass in males in our prebiotic group with a trend towards a decrease in ABT + PRE group. Furthermore, intestinal expression of tight junction proteins was normalized with prebiotic co-administration (ABT + PRE) in males. Interestingly, these health-promoting effects of OFS supplementation were abrogated once *Lactobacillus* and *Bifidobacterium* growth was inhibited with antibiotics [17]. It is possible that the positive effects of OFS supplementation in our study was a consequence of increased levels of *Lactobacillaceae*, which were depleted after PAT. In line with this, other studies showed that probiotic strains such as *Lactobacillus rhamnosus* GG and *Lactobacillus casei* DN-114-001 improve epithelial barrier function via tight junction proteins [57,58]. In addition, prebiotics also improve gut permeability due to changes in the morphology of the intestine by increasing the villus height and crypt depth and thickening the mucus layer [56]. During the fermentation of prebiotics, butyrate is produced, feeding colonocytes and increasing the mucus layer [56]. Increased cecum size is a marker of increased bacterial fermentation [17,59] which was clearly evident in our prebiotic animals in both males and females. Taken together, we believe improvements in insulin sensitivity in our prebiotic groups might be partially explained by increased levels of *Lactobacillaceae*, normalization of extremely high levels of *Bacteroidaceae* and improved gut permeability seen by lower LPS levels and higher expression of tight junction proteins in prebiotic groups. Although *Bacteroidaceae* represent normal commensals in the gut, under certain conditions they have been shown to cause substantial inflammatory distress [60,61]. Certain species, such as *Bacteroides fragilis* contain a unique LPS whose presence in host serum is a major contributor to systemic inflammation in part via intestinal barrier-disrupting proteolytic endotoxins [62,63]. Future in-depth analysis of the *Bacteroidaceae* species in our animals as well as use of a functional intestinal permeability test such as FITC-4000 would be valuable in confirming our suggestive findings.

Besides gut microbiota/body composition/metabolic changes, prebiotics also altered the expression of select genes in the liver and hypothalamus. Specifically, reductions in hypothalamic *IRS-1/2* gene expression seen in PAT males were normalized with prebiotics and could further explain improvements in insulin sensitivity seen in prebiotic males. Of note, it was shown previously [59] that prebiotic can modify hypothalamic expression of genes relevant to anxiety and the authors proposed that changes were mediated by SCFAs. To our knowledge, we are the first to show that early life prebiotic supplementation added to PAT alters the expression of a select number of hypothalamic genes but is unable to correct hepatic *IRS* expression in males. Nevertheless, since hypothalamic IR expression plays a major role in energy homeostasis (IR deficiency led to increased body weight and adiposity [64]), we hypothesize that increasing hypothalamic IRS with prebiotics could promoted leanness in our study, at least in part. Given the limited number of genes examined in the liver and hypothalamus and the fact that we did not examine protein expression, suggests that more in-depth examination of the effects of prebiotic co-administration are needed.

Taken together, we show that direct pulsed administration of azithromycin to young rats impairs microbiota composition/maturation, body weight, fat mass, serum LPS, hepatic/ileal/hypothalamic gene expression and insulin production/sensitivity in a sex-specific manner. Importantly, we demonstrate that the unfavorable outcomes of antibiotics are prevented with prebiotic co-administration even when the doses administered are therapeutic and mimic administration of antibiotics in pediatric populations. It is unknown whether the benefits of this non-invasive intervention with prebiotics translates to human children, but the potential to reduce harm of early life antibiotic exposure is promising and warrants further investigation.

## Data Availability

The data set is available at NCBI SRA accession: PRJNA641149.

## Supplementary Materials

The following are available online at https://www.mdpi.com/2227-9059/9/1/66/s1. Supplemental Table S1: Primer sequences for RT-qPCR. Supplemental Table S2: Between-group changes in relative abundance of the 15 most abundant bacterial families in male rats after the first, second, third antibiotic pulse and at the end of the study. (*n* = 8–10 rats per group/time point). Supplemental Table S3: Between-group changes in relative abundance of the 15 most abundant bacterial families in female rats after the first, second, third antibiotic pulse and at the end of the study. (*n* = 8–10 rats per group/time point). Supplemental Figure S1: ABT male offspring display increased endotoxemia at the end of the study. (A and B) Circulating LPS levels in males at (A) week 7 of life and (B) end of the study (*n* = 8–10 rats per group/time point). (C,D) Circulating LPS levels in females at (C) week 7 of life and (D) end of the study (*n* = 8–10 rats per group/time point). Different superscript letters denote significant differences between the groups, *p* < 0.05. CTR, control; ATB, antibiotic; PRE, prebiotic; ATB + PRE, antibiotic + prebiotic; LEAN, lean control.

## References

[1] Reinhardt C., Reigstad C.S., Bäckhed F. (2009). Intestinal microbiota during infancy and its implications for obesity. J. Pediatr. Gastroenterol. Nutr..

[2] Turnbaugh P.J., Ley R.E., Mahowald M.A., Magrini V., Mardis E.R., Gordon J.I. (2006). An obesity-associated gut microbiome with increased capacity for energy harvest. Nature.

[3] Arrieta M.-C., Stiemsma L.T., Amenyogbe N., Brown E.M., Finlay B. (2014). The intestinal microbiome in early Life: Health and disease. Front. Immunol..

[4] Clavenna A., Bonati M. (2011). Differences in antibiotic prescribing in paediatric outpatients. Arch. Dis. Child..

[5] Maukonen J., Saarela M. (2015). Human gut microbiota: Does diet matter?. Proc. Nutr. Soc..

[6] Pallister T., Jackson M.A., Martin T.C., Glastonbury C.A., Jennings A., Beaumont M., Mohney R.P., Small K.S., MacGregor A., Steves C.J. (2017). Untangling the relationship between diet and visceral fat mass through blood metabolomics and gut microbiome profiling. Int. J. Obes..

[7] Nakayama J., Watanabe K., Jiang J., Matsuda K., Chao S.-H., Haryono P., La-ongkham O., Sarwoko M.-A., Sujaya I.N., Zhao L. (2015). Diversity in gut bacterial community of school-age children in Asia. Sci. Rep..

[8] Renz H., Brandtzaeg P., Hornef M. (2012). The impact of perinatal immune development on mucosal homeostasis and chronic inflammation. Nat. Rev. Immunol..

[9] West C.E., Jenmalm M.C., Prescott S.L. (2015). The gut microbiota and its role in the development of allergic disease: A wider perspective. Clin. Exp. Allergy.

[10] Nobel Y.R., Cox L.M., Kirigin F.F., Bokulich N.A., Yamanishi S., Teitler I., Chung J., Sohn J., Barber C.M., Goldfarb D.S. (2015). Metabolic and metagenomic outcomes from early-life pulsed antibiotic treatment. Nat. Commun.

[11] Taylor J.H., Gordon W.S. (1955). Growth-promoting activity for pigs of inactivated penicillin. Nature.

[12] Cox L.M., Yamanishi S., Sohn J., Alekseyenko A.V., Leung J.M., Cho I., Kim S.G., Li H., Gao Z., Mahana D. (2014). Altering the intestinal microbiota during a critical developmental window has lasting metabolic consequences. Cell.

[13] Klancic T., Laforest-Lapointe I., Choo A., Nettleton J.E., Chleilat F., Noye Tuplin E., Alukic E., Cho N.A., Nicolucci A.C., Arrieta M.-C. (2020). Prebiotic oligofructose prevents antibiotic-induced obesity risk and improve metabolic and gut microbiota profiles in rat dams and offspring. Mol. Nutr. Food Res..

[14] Gibson G.R., Hutkins R., Sanders M.E., Prescott S.L., Reimer R.A., Salminen S.J., Scott K., Stanton C., Swanson K.S., Cani P.D. (2017). Expert consensus document: The international scientific association for probiotics and prebiotics (ISAPP) consensus statement on the definition and scope of prebiotics. Nat. Rev. Gastroenterol. Hepatol..

[15] Nicolucci A.C., Hume M.P., Martínez I., Mayengbam S., Walter J., Reimer R.A. (2017). Prebiotics reduce body fat and alter intestinal microbiota in children who are overweight or with obesity. Gastroenterology.

[16] Paul H.A., Collins K.H., Nicolucci A.C., Urbanski S.J., Hart D.A., Vogel H.J., Reimer R.A. (2019). Maternal prebiotic supplementation reduces fatty liver development in offspring through altered microbial and metabolomic profiles in rats. FASEB J..

[17] Bomhof M.R., Paul H.A., Geuking M.B., Eller L.K., Reimer R.A. (2016). Improvement in adiposity with oligofructose is modified by antibiotics in obese rats. FASEB J..

[18] Durkin M.J., Jafarzadeh S.R., Hsueh K., Sallah Y.H., Munshi K.D., Henderson R.R., Fraser V.J. (2018). Outpatient antibiotic prescription trends in the United States: A national cohort study. Infect. Control. Hosp. Epidemiol..

[19] Shepard R.M., Falkner F.C. (1990). Pharmacokinetics of azithromycin in rats and dogs. J. Antimicrob. Chemother..

[20] Azithromycin 200 mg/5 mL Powder for Oral Suspension-Summary of Product Characteristics (SmPC)-(eMC). https://www.medicines.org.uk/emc/medicine/22608.

[21] Kaliannan K., Wang B., Li X.-Y., Bhan A.K., Kang J.X. (2016). Omega-3 fatty acids prevent early-life antibiotic exposure-induced gut microbiota dysbiosis and later-life obesity. Int. J. Obes..

[22] Cani P.D., Dewever C., Delzenne N.M. (2004). Inulin-type fructans modulate gastrointestinal peptides involved in appetite regulation (glucagon-like peptide-1 and ghrelin) in rats. Br. J. Nutr..

[23] Parnell J.A., Reimer R.A. (2012). Prebiotic fibres dose-dependently increase satiety hormones and alter bacteroidetes and firmicutes in lean and obese JCR: LA-cp rats. Br. J. Nutr..

[24] Delzenne N.M. (2003). Oligosaccharides: State of the art. Proc. Nutr. Soc..

[25] Ruiz V.E., Battaglia T., Kurtz Z.D., Bijnens L., Ou A., Engstrand I., Zheng X., Iizumi T., Mullins B.J., Müller C.L. (2017). A single early-in-life macrolide course has lasting effects on murine microbial network topology and immunity. Nat. Commun..

[26] Bomhof M.R., Saha D.C., Reid D.T., Paul H.A., Reimer R.A. (2014). Combined effects of oligofructose and *Bifidobacterium animalis* on gut microbiota and glycemia in obese rats: Combined prebiotic and probiotic in obesity. Obesity.

[27] Cersosimo E., Solis-Herrera C., Trautmann E.M., Malloy J.L., Triplitt C. (2014). Assessment of pancreatic β-cell function: Review of methods and clinical applications. Curr. Diabetes Rev..

[28] Parnell J.A., Reimer R.A. (2010). Effect of prebiotic fibre supplementation on hepatic gene expression and serum lipids: A dose–response study in JCR:LA-cp rats. Br. J. Nutr..

[29] Parnell J.A., Reimer R.A. (2008). Differential secretion of satiety hormones with progression of obesity in JCR: LA-corpulent rats. Obesity.

[30] Callahan B.J., McMurdie P.J., Rosen M.J., Han A.W., Johnson A.J.A., Holmes S.P. (2016). DADA2: High resolution sample inference from Illumina amplicon data. Nat. Methods.

[31] McMurdie P.J., Holmes S. (2013). Phyloseq: An R package for reproducible interactive analysis and graphics of microbiome census data. PLoS ONE.

[32] McMurdie P.J., Holmes S. (2014). Waste not, want not: Why rarefying microbiome data is inadmissible. PLoS Comput. Biol..

[33] Anders S., Huber W. (2010). Differential expression analysis for sequence count data. Genome Biol..

[34] Cho I., Yamanishi S., Cox L., Methé B.A., Zavadil J., Li K., Gao Z., Mahana D., Raju K., Teitler I. (2012). Antibiotics in early life alter the murine colonic microbiome and adiposity. Nature.

[35] Velasquez-Manoff M. (2015). Gut microbiome: The peacekeepers. Nature.

[36] Iozzo P., Sanguinetti E. (2018). Early dietary patterns and microbiota development: Still a way to go from descriptive interactions to health-relevant solutions. Front. Nutr..

[37] Hersh A.L., Shapiro D.J., Pavia A.T., Shah S.S. (2011). Antibiotic prescribing in ambulatory pediatrics in the United States. Pediatrics.

[38] Ajslev T.A., Andersen C.S., Gamborg M., Sørensen T.I.A., Jess T. (2011). Childhood overweight after establishment of the gut microbiota: The role of delivery mode, pre-pregnancy weight and early administration of antibiotics. Int. J. Obes..

[39] Azad M.B., Bridgman S.L., Becker A.B., Kozyrskyj A.L. (2014). Infant antibiotic exposure and the development of childhood overweight and central adiposity. Int. J. Obes..

[40] Bailey L.C., Forrest C.B., Zhang P., Richards T.M., Livshits A., DeRusso P.A. (2014). Association of antibiotics in infancy with early childhood obesity. JAMA Pediatr..

[41] Murphy R., Stewart A.W., Braithwaite I., Beasley R., Hancox R.J., Mitchell E.A. (2014). Antibiotic treatment during infancy and increased body mass index in boys: An international cross-sectional study. Int. J. Obes..

[42] Saari A., Virta L.J., Sankilampi U., Dunkel L., Saxen H. (2015). Antibiotic exposure in infancy and risk of being overweight in the first 24 months of life. Pediatrics.

[43] Cox L.M., Blaser M.J. (2015). Antibiotics in early life and obesity. Nat. Rev. Endocrinol..

[44] Beltrand J., Soboleva T.K., Shorten P.R., Derraik J.G.B., Hofman P., Albertsson-Wikland K., Hochberg Z., Cutfield W.S. (2012). Post-term birth is associated with greater risk of obesity in adolescent males. J. Pediatr..

[45] Gabory A., Roseboom T.J., Moore T., Moore L.G., Junien C. (2013). Placental contribution to the origins of sexual dimorphism in health and diseases: Sex chromosomes and epigenetics. Biol Sex Differ..

[46] Leong K.S.W., Derraik J.G.B., Hofman P.L., Cutfield W.S. (2018). Antibiotics, gut microbiome and obesity. Clin. Endocrinol..

[47] Ding S., Lund P.K. (2011). Role of intestinal inflammation as an early event in obesity and insulin resistance. Curr. Opin. Clin. Nutr. Metab. Care.

[48] Ghanim H., Abuaysheh S., Sia C.L., Korzeniewski K., Chaudhuri A., Fernandez-Real J.M., Dandona P. (2009). Increase in plasma endotoxin concentrations and the expression of Toll-like receptors and suppressor of cytokine signaling-3 in mononuclear cells after a high-fat, high-carbohydrate meal: Implications for insulin resistance. Diabetes Care.

[49] D’Aversa F., Tortora A., Ianiro G., Ponziani F.R., Annicchiarico B.E., Gasbarrini A. (2013). Gut microbiota and metabolic syndrome. Intern. Emerg Med..

[50] Atli O., Ilgin S., Altuntas H., Burukoglu D. (2015). Evaluation of azithromycin induced cardiotoxicity in rats. Int. J. Clin. Exp. Med..

[51] Sommer F., Anderson J.M., Bharti R., Raes J., Rosenstiel P. (2017). The resilience of the intestinal microbiota influences health and disease. Nat. Rev. Microbiol..

[52] Osaka T., Moriyama E., Arai S., Date Y., Yagi J., Kikuchi J., Tsuneda S. (2017). Meta-analysis of fecal microbiota and metabolites in experimental colitic mice during the inflammatory and healing phases. Nutrients.

[53] Vieira-Silva S., Falony G., Darzi Y., Lima-Mendez G., Garcia Yunta R., Okuda S., Vandeputte D., Valles-Colomer M., Hildebrand F., Chaffron S. (2016). Species–function relationships shape ecological properties of the human gut microbiome. Nat. Microbiol..

[54] Boursi B., Mamtani R., Haynes K., Yang Y.-X. (2015). The effect of past antibiotic exposure on diabetes risk. Eur. J. Endocrinol..

[55] Blaser M.J. (2016). Antibiotic use and its consequences for the normal microbiome. Science.

[56] Cani P.D., Possemiers S., van de Wiele T., Guiot Y., Everard A., Rottier O., Geurts L., Naslain D., Neyrinck A., Lambert D.M. (2009). Changes in gut microbiota control inflammation in obese mice through a mechanism involving GLP-2-driven improvement of gut permeability. Gut.

[57] Johnson-Henry K.C., Donato K.A., Shen-Tu G., Gordanpour M., Sherman P.M. (2008). Lactobacillus rhamnosus strain GG prevents enterohemorrhagic Escherichia coli O157: H7-induced changes in epithelial barrier function. Infect. Immun..

[58] Parassol N., Freitas M., Thoreux K., Dalmasso G., Bourdet-Sicard R., Rampal P. (2005). Lactobacillus casei DN-114 001 inhibits the increase in paracellular permeability of enteropathogenic *Escherichia coli*-infected T84 cells. Res. Microbiol..

[59] Burokas A., Arboleya S., Moloney R.D., Peterson V.L., Murphy K., Clarke G., Stanton C., Dinan T.G., Cryan J.F. (2017). Targeting the microbiota-gut-brain axis: Prebiotics have anxiolytic and antidepressant-like effects and reverse the impact of chronic stress in mice. Biol. Psychiatry.

[60] Fathi P., Wu S. (2016). Isolation, detection, and characterization of enterotoxigenic bacteroides fragilis in clinical samples. Open Microbiol. J..

[61] Hofer U. (2014). Microbiome: B. fragilis and the brain. Nat. Rev. Microbiol..

[62] Choi V.M., Herrou J., Hecht A.L., Teoh W.P., Turner J.R., Crosson S., Bubeck Wardenburg J. (2016). Activation of *Bacteroides fragilis* toxin by a novel bacterial protease contributes to anaerobic sepsis in mice. Nat. Med..

[63] Lukiw W.J. (2016). *Bacteroides fragilis Lipopolysaccharide* and Inflammatory Signaling in Alzheimer’s Disease. Front. Microbiol..

[64] Loh K., Zhang L., Brandon A., Wang Q., Begg D., Qi Y., Fu M., Kulkarni R., Teo J., Baldock P. (2017). Insulin controls food intake and energy balance via NPY neurons. Mol. Metab..

