# Supplementary material for: Concurrent Prebiotic Intake Reverses Insulin Resistance Induced by Early-Life Pulsed Antibiotic in Rats
Source: Biomedicines. 2021 Jan 12;9(1):66. doi: 10.3390/biomedicines9010066 (PMC7827688; doi:10.3390/biomedicines9010066)
Supplement: Supplementary file 1 [file biomedicines-09-00066-s001.pdf]

Supplemental Table 1: Primer sequences for RT-qPCR

Gene	Forward Primer (5'-3')	Reverse Primer (5'-3')
**Liver, Ileum and Hypothalamus**		
IRS-1	CTCTGCTTCTGCTTCTGTTAC	TGGTTATGGTTGGGACTTAGG
IRS-2	AAGATAGCGGGTACATGCGAAT	GCAGCTTAGGGTCTGGGTTCT
ZO-1	AAAGGTGAAACTCTGCTGAG	GATCTCCAGGAAGACACTTG
Occludin	GAGGACTGGCTCAGGGAATATC	TTGTTGACCTCGTCGAGTTCTG
Muc2	CCACCATTACCACCACCTCAG	CGATCACCACCATTGCCATTG
MMP9	CATGCGCTGGGCTTAGATCA	GAGGCCTTGGGTCAGGTTTAGAG
IL-10	AGTCAGCCAGACCCACATG	GCAACCCAAGTAACCCTTAAAG
POMC	AGGTTAAGGAGCAGTGACTAAG	AGCAGAATCTCGGCATCTTC
AGRP	CGTGCTACTGCCGCTTCTTC	CCCTGCCTTTGCCCAACATC
NPY	GCTCGTGTGTTTGGGCATTC	GAGATTGATGTAGTGTCGCAGAG
β-Actin	ATCAAGATCATTGCTCCTCCTG	GACTCATCGTACTCCTGCTTG
18S	TGACTCAACACGGGAAACC	TCGCTCCACCAACTAAGAAC
GAPDH	CAAGTTCAACGGCACAGTCAAG	ACATACTCAGCACCAGCATCAC

Supplemental Table 2: Between-group changes in relative abundance of the 15 most abundant bacterial families in male rats after the first, second, third antibiotic pulse and at the end of the study. (n=8-10 rats per group/time point).

- After first antibiotic exposure

Family/Group	CTR	ABT	PRE	ABT+PRE	LEAN
Peptococcaceae	0.002±0.000^{a}	0.000±0.000^{b}	0.000±0.000^{b}	0.000±0.000^{b}	0.002±0.000^{a}
Bifidobacteriaceae	0.000±0.000^{a}	0.000±0.000^{a}	0.004±0.002^{b}	0.000±0.000^{a}	0.000±0.000^{a}
Verrucomicrobiaceae	0.030±0.008^{a}	0.000±0.000^{b}	0.006±0.005^{b}	0.000±0.000^{b}	0.038±0.010^{a}
Enterococcaceae	0.001±0.000^{a}	0.046±0.010^{bc}	0.004±0.001^{ac}	0.261±0.022^{b}	0.000±0.000^{a}
Porphyromonadaceae	0.043±0.010^{a}	0.000±0.000^{b}	0.016±0.005^{ac}	0.006±0.004^{bc}	0.014±0.006^{ac}
Prevotellaceae	0.068±0.017^{a}	0.000±0.000^{b}	0.001±0.001^{b}	0.000±0.000^{b}	0.118±0.019^{a}
Erysipelotrichaceae	0.021±0.006^{a}	0.002±0.000^{b}	0.066±0.019^{a}	0.001±0.001^{b}	0.015±0.003^{a}
Enterobacteriaceae	0.014±0.003^{a}	0.018±0.008^{a}	0.175±0.031^{b}	0.205±0.025^{b}	0.016±0.004^{a}
Ruminococcaceae	0.051±0.007^{a}	0.035±0.005^{a}	0.001±0.001^{b}	0.002±0.001^{b}	0.063±0.011^{a}
Bacteroidales_S24-7_group	0.121±0.019^{a}	0.003±0.002^{b}	0.003±0.002^{b}	0.000±0.000^{b}	0.121±0.008^{a}
Peptostreptococcaceae	0.041±0.008^{a}	0.003±0.001^{b}	0.033±0.009^{a}	0.000±0.000^{b}	0.059±0.010^{a}
Clostridiaceae_1	0.011±0.003^{a}	0.000±0.000^{b}	0.044±0.011^{a}	0.000±0.000^{b}	0.010±0.002^{a}
Lachnospiraceae	0.187±0.036^{ab}	0.203±0.017^{a}	0.120±0.010^{b}	0.196±0.022^{ab}	0.130±0.019^{b}
Bacteroidaceae	0.071±0.016^{b}	0.605±0.027^{a}	0.126±0.026^{bc}	0.217±0.041^{ac}	0.066±0.029^{b}
Lactobacillaceae	0.323±0.042^{a}	0.081±0.012^{b}	0.400±0.025^{a}	0.111±0.025^{b}	0.337±0.049^{a}

- After second antibiotic exposure – Males

Family/Group	CTR	ABT	PRE	ABT+PRE	LEAN
Peptococcaceae	0.003±0.001^{a}	0.001±0.001^{b}	0.000±0.000^{b}	0.000±0.000^{b}	0.002±0.001^{a}
Bifidobacteriaceae	0.002±0.000^{acd}	0.000±0.000^{b}	0.039±0.019^{c}	0.000±0.000^{b}	0.001±0.000^{bd}
Verrucomicrobiaceae	0.045±0.014^{a}	0.018±0.014^{a}	0.000±0.000^{b}	0.000±0.000^{b}	0.003±0.001^{ab}
Enterococcaceae	0.000±0.000^{a}	0.018±0.006^{bc}	0.003±0.001^{ac}	0.088±0.029^{b}	0.001±0.001^{a}
Porphyromonadaceae	0.096±0.010^{a}	0.000±0.000^{b}	0.021±0.005^{c}	0.105±0.021^{a}	0.004±0.001^{bc}
Prevotellaceae	0.065±0.015^{a}	0.000±0.000^{b}	0.000±0.000^{b}	0.000±0.000^{b}	0.187±0.006^{a}
Erysipelotrichaceae	0.019±0.004^{a}	0.020±0.016^{a}	0.206±0.025^{b}	0.062±0.035^{a}	0.022±0.002^{a}
Enterobacteriaceae	0.003±0.002^{a}	0.000±0.000^{a}	0.121±0.016^{bc}	0.231±0.037^{b}	0.003±0.000^{ac}
Ruminococcaceae	0.033±0.006^{ac}	0.077±0.015^{a}	0.002±0.000^{b}	0.017±0.006^{bc}	0.086±0.015^{a}
Bacteroidales_S24-7_group	0.123±0.021^{a}	0.012±0.004^{b}	0.000±0.000^{c}	0.000±0.000^{c}	0.131±0.007^{a}
Peptostreptococcaceae	0.089±0.008^{a}	0.011±0.007^{bc}	0.012±0.005^{bc}	0.003±0.003^{c}	0.029±0.008^{ab}
Clostridiaceae_1	0.129±0.014^{a}	0.021±0.012^{b}	0.152±0.050^{a}	0.037±0.016^{b}	0.011±0.005^{b}
Lachnospiraceae	0.120±0.024^{ac}	0.291±0.026^{b}	0.065±0.025^{a}	0.224±0.024^{b}	0.181±0.019^{bc}
Bacteroidaceae	0.043±0.010^{ac}	0.477±0.024^{b}	0.142±0.022^{a}	0.181±0.040^{a}	0.010±0.003^{c}
Lactobacillaceae	0.215±0.037^{a}	0.051±0.019^{b}	0.230±0.036^{a}	0.051±0.050^{b}	0.315±0.026^{a}

- After third antibiotic exposure – Males

Family/Group	CTR	ABT	PRE	ABT+PRE	LEAN
Peptococcaceae	0.002±0.001^{a}	0.000±0.000^{b}	0.000±0.000^{b}	0.000±0.000^{b}	0.003±0.000^{a}
Bifidobacteriaceae	0.002±0.001^{a}	0.000±0.000^{a}	0.338±0.015^{b}	0.000±0.000^{a}	0.001±0.000^{a}
Verrucomicrobiaceae	0.030±0.012^{a}	0.009±0.005^{a}	0.000±0.000^{b}	0.000±0.000^{b}	0.000±0.000^{b}

Enterococcaceae	0.000±0.000^{a}	0.011±0.006^{b}	0.010±0.003^{b}	0.039±0.008^{b}	0.000±0.000^{a}
Porphyromonadaceae	0.083±0.014^{a}	0.000±0.000^{b}	0.001±0.001^{bd}	0.015±0.012^{bc}	0.006±0.001^{acd}
Prevotellaceae	0.031±0.007^{a}	0.000±0.000^{b}	0.000±0.000^{b}	0.000±0.000^{b}	0.150±0.018^{a}
Erysipelotrichaceae	0.007±0.002^{a}	0.038±0.004^{bc}	0.041±0.011^{bc}	0.134±0.047^{b}	0.016±0.002^{ac}
Enterobacteriaceae	0.002±0.000^{a}	0.000±0.000^{a}	0.022±0.008^{b}	0.026±0.011^{b}	0.001±0.000^{a}
Ruminococcaceae	0.029±0.009^{ad}	0.069±0.011^{ac}	0.000±0.000^{b}	0.003±0.001^{bd}	0.120±0.13^{c}
Bacteroidales_S24-7_group	0.131±0.023^{a}	0.021±0.005^{a}	0.000±0.000^{b}	0.000±0.000^{b}	0.132±0.017^{a}
Peptostreptococcaceae	0.104±0.012^{a}	0.021±0.015^{bc}	0.001±0.001^{b}	0.003±0.002^{b}	0.052±0.012^{ac}
Clostridiaceae_1	0.149±0.024^{a}	0.030±0.015^{b}	0.031±0.017^{b}	0.214±0.058^{a}	0.041±0.014^{b}
Lachnospiraceae	0.079±0.024^{ac}	0.249±0.014^{b}	0.008±0.005^{a}	0.017±0.010^{a}	0.165±0.011^{bc}
Bacteroidaceae	0.030±0.007^{a}	0.452±0.029^{b}	0.017±0.007^{a}	0.049±0.033^{a}	0.006±0.002^{a}
Lactobacillaceae	0.307±0.036^{a}	0.092±0.036^{b}	0.530±0.031^{c}	0.499±0.082^{ac}	0.300±0.037^{a}

- End of Study – Males

Family/Group	CTR	ABT	PRE	ABT+PRE	LEAN
Peptococcaceae	0.004±0.000	0.006±0.001	0.004±0.001	0.004±0.000	0.003±0.000
Bifidobacteriaceae	0.000±0.000^{a}	0.000±0.000^{a}	0.000±0.000^{a}	0.000±0.000^{a}	0.000±0.000^{a}
Verrucomicrobiaceae	0.005±0.004^{a}	0.079±0.013^{b}	0.050±0.009^{b}	0.060±0.015^{b}	0.000±0.000^{a}
Enterococcaceae	0.000±0.000^{a}	0.001±0.000^{a}	0.005±0.003^{a}	0.001±0.000^{a}	0.000±0.000^{a}
Porphyromonadaceae	0.023±0.003^{ac}	0.051±0.008^{b}	0.037±0.005^{ab}	0.034±0.007^{ab}	0.007±0.001^{c}
Prevotellaceae	0.019±0.004^{a}	0.000±0.000^{b}	0.000±0.000^{b}	0.001±0.000^{b}	0.130±0.012^{a}
Erysipelotrichaceae	0.015±0.006^{a}	0.017±0.004^{a}	0.040±0.010^{a}	0.015±0.003^{a}	0.032±0.013^{a}
Enterobacteriaceae	0.001±0.000^{ac}	0.003±0.000^{b}	0.002±0.000^{ab}	0.002±0.000^{ab}	0.000±0.000^{c}
Ruminococcaceae	0.040±0.005^{a}	0.071±0.011^{a}	0.046±0.007^{a}	0.054±0.011^{a}	0.147±0.018^{b}
Bacteroidales_S24-7_group	0.138±0.013^{a}	0.015±0.002^{b}	0.021±0.010^{b}	0.022±0.004^{b}	0.136±0.021^{a}
Peptostreptococcaceae	0.144±0.013^{a}	0.084±0.012^{bc}	0.172±0.012^{a}	0.132±0.017^{ac}	0.080±0.019^{bc}
Clostridiaceae_1	0.198±0.016^{a}	0.174±0.034^{a}	0.226±0.020^{a}	0.140±0.033^{a}	0.019±0.007^{b}
Lachnospiraceae	0.078±0.006^{a}	0.135±0.017^{ab}	0.109±0.014^{ab}	0.113±0.018^{ab}	0.178±0.026^{b}
Bacteroidaceae	0.017±0.004^{ad}	0.172±0.022^{b}	0.064±0.011^{cd}	0.128±0.011^{bc}	0.008±0.002^{a}
Lactobacillaceae	0.294±0.040	0.183±0.020	0.210±0.037	0.189±0.046	0.249±0.043

Values displayed are mean±SE (relative abundance). Kruskal-Wallis test with Dunn post-hoc tests and Benjamin-Holmes False Discovery Rate (FDR) correction; $P<0.05$. CTR, control; ABT, antibiotic; PRE, prebiotic; ABT+PRE, antibiotic+prebiotic; LEAN, lean control.

Supplemental Table 3: Between-group changes in relative abundance of the 15 most abundant bacterial families in female rats after the first, second, third antibiotic pulse and at the end of the study. (n=8-10 rats per group/time point).

- After first antibiotic exposure – Females

Family/Group	CTR	ABT	PRE	ABT+PRE	LEAN
Peptococcaceae	0.004±0.001^{a}	0.000±0.000^{b}	0.000±0.000^{b}	0.000±0.000^{b}	0.003±0.001^{a}
Bifidobacteriaceae	0.000±0.000^{a}	0.000±0.000^{a}	0.022±0.011^{b}	0.000±0.000^{a}	0.000±0.000^{a}
Enterococcaceae	0.004±0.002^{a}	0.063±0.011^{b}	0.002±0.001^{a}	0.229±0.035^{b}	0.001±0.000^{a}
Verrucomicrobiaceae	0.056±0.017^{a}	0.001±0.000^{bd}	0.010±0.004^{acd}	0.000±0.000^{b}	0.030±0.011^{c}
Porphyromonadaceae	0.037±0.008^{a}	0.000±0.000^{b}	0.018±0.007^{ac}	0.006±0.003^{bc}	0.005±0.002^{bc}
Enterobacteriaceae	0.014±0.003^{a}	0.014±0.005^{a}	0.126±0.033^{b}	0.193±0.030^{b}	0.008±0.002^{a}
Erysipelotrichaceae	0.021±0.004^{a}	0.001±0.001^{b}	0.078±0.013^{a}	0.008±0.007^{b}	0.009±0.001^{b}
Prevotellaceae	0.065±0.013^{a}	0.000±0.000^{b}	0.001±0.001^{b}	0.000±0.000^{b}	0.131±0.010^{a}
Ruminococcaceae	0.064±0.009^{a}	0.024±0.004^{ab}	0.003±0.003^{b}	0.003±0.002^{b}	0.068±0.011^{a}
Peptostreptococcaceae	0.050±0.008^{a}	0.002±0.000^{b}	0.036±0.010^{a}	0.001±0.001^{b}	0.046±0.008^{a}
Bacteroidales_S24-7_group	0.153±0.017^{a}	0.007±0.003^{b}	0.008±0.003^{b}	0.000±0.000^{b}	0.134±0.010^{a}
Clostridiaceae_1	0.048±0.024^{a}	0.000±0.000^{b}	0.022±0.005^{a}	0.000±0.000^{b}	0.0122±0.003^{a}
Bacteroidaceae	0.070±0.009^{a}	0.548±0.031^{b}	0.117±0.023^{ac}	0.208±0.019^{bc}	0.016±0.009^{a}
Lachnospiraceae	0.168±0.019^{a}	0.189±0.013^{a}	0.132±0.016^{a}	0.162±0.024^{a}	0.143±0.013^{a}
Lactobacillaceae	0.232±0.023^{a}	0.147±0.036^{a}	0.419±0.032^{b}	0.188±0.050^{a}	0.383±0.025^{b}

- After second antibiotic exposure – Females

Family/Group	CTR	ABT	PRE	ABT+PRE	LEAN
Peptococcaceae	0.004±0.001^{a}	0.000±0.000^{b}	0.000±0.000^{b}	0.000±0.000^{b}	0.004±0.001^{a}
Bifidobacteriaceae	0.001±0.000^{a}	0.000±0.000^{a}	0.071±0.028^{b}	0.000±0.000^{a}	0.000±0.000^{a}
Enterococcaceae	0.001±0.001^{ac}	0.009±0.004^{b}	0.003±0.001^{bc}	0.059±0.016^{b}	0.000±0.000^{a}
Verrucomicrobiaceae	0.060±0.017^{a}	0.015±0.012^{b}	0.000±0.000^{b}	0.000±0.000^{b}	0.001±0.001^{b}
Porphyromonadaceae	0.089±0.010^{a}	0.000±0.000^{b}	0.019±0.004^{c}	0.149±0.023^{a}	0.005±0.001^{bc}
Enterobacteriaceae	0.005±0.002^{ac}	0.000±0.000^{a}	0.094±0.014^{bc}	0.215±0.036^{b}	0.002±0.001^{a}
Erysipelotrichaceae	0.022±0.004^{a}	0.011±0.003^{a}	0.195±0.035^{b}	0.083±0.045^{a}	0.015±0.035^{a}
Prevotellaceae	0.056±0.015^{a}	0.000±0.000^{b}	0.000±0.000^{b}	0.000±0.000^{b}	0.191±0.009^{a}
Ruminococcaceae	0.032±0.004^{ac}	0.075±0.011^{a}	0.002±0.001^{b}	0.013±0.005^{bc}	0.087±0.008^{a}
Peptostreptococcaceae	0.089±0.006^{a}	0.030±0.026^{b}	0.009±0.003^{bc}	0.000±0.000^{c}	0.038±0.013^{ab}
Bacteroidales_S24-7_group	0.120±0.014^{ac}	0.009±0.003^{ab}	0.000±0.000^{b}	0.000±0.000^{b}	0.153±0.010^{c}
Clostridiaceae_1	0.125±0.021^{a}	0.026±0.014^{b}	0.144±0.022^{a}	0.078±0.034^{ab}	0.017±0.009^{b}
Bacteroidaceae	0.068±0.018^{ac}	0.477±0.029^{b}	0.131±0.067^{c}	0.152±0.043^{c}	0.009±0.002^{a}
Lachnospiraceae	0.109±0.017^{a}	0.270±0.017^{b}	0.064±0.016^{a}	0.238±0.031^{b}	0.135±0.016^{a}
Lactobacillaceae	0.203±0.002^{ac}	0.067±0.016^{bc}	0.262±0.056^{a}	0.012±0.004^{b}	0.3270.018^{a}

- After third antibiotic exposure – Females

Family/Group	CTR	ABT	PRE	ABT+PRE	LEAN
Peptococcaceae	0.003±0.001^{a}	0.000±0.000^{b}	0.000±0.000^{b}	0.000±0.000^{b}	0.002±0.000^{a}
Bifidobacteriaceae	0.001±0.001^{a}	0.000±0.000^{a}	0.308±0.047^{b}	0.000±0.000^{a}	0.000±0.000^{a}

Enterococcaceae	0.000±0.000^{a}	0.001±0.000^{ab}	0.011±0.003^{bc}	0.050±0.0131^{c}	0.000±0.000^{a}
Verrucomicrobiaceae	0.068±0.027^{a}	0.035±0.015^{a}	0.000±0.000^{b}	0.000±0.000^{b}	0.001±0.001^{b}
Porphyromonadaceae	0.093±0.022^{a}	0.000±0.000^{b}	0.001±0.001^{b}	0.001±0.000^{b}	0.007±0.001^{a}
Enterobacteriaceae	0.006±0.002^{bc}	0.000±0.000^{a}	0.024±0.006^{b}	0.004±0.001^{ab}	0.001±0.000^{ac}
Erysipelotrichaceae	0.008±0.001^{a}	0.029±0.006^{a}	0.112±0.026^{a}	0.092±0.016^{a}	0.008±0.002^{a}
Prevotellaceae	0.036±0.003^{a}	0.000±0.000^{b}	0.000±0.000^{b}	0.000±0.000^{b}	0.182±0.010^{a}
Ruminococcaceae	0.028±0.006^{ab}	0.1503±0.015^{a}	0.001±0.000^{b}	0.004±0.001^{b}	0.094±0.016^{a}
Peptostreptococcaceae	0.127±0.015^{a}	0.011±0.008^{b}	0.014±0.006^{bc}	0.002±0.001^{b}	0.050±0.017^{ac}
Bacteroidales_S24-7_group	0.123±0.019^{ab}	0.014±0.002^{b}	0.000±0.000^{c}	0.000±0.000^{c}	0.150±0.007^{a}
Clostridiaceae_1	0.138±0.019^{ac}	0.018±0.013^{b}	0.112±0.038^{ad}	0.493±0.056^{c}	0.019±0.012^{bd}
Bacteroidaceae	0.030±0.009^{a}	0.459±0.017^{b}	0.016±0.010^{a}	0.013±0.005^{a}	0.008±0.002^{a}
Lachnospiraceae	0.073±0.008^{ac}	0.245±0.019^{b}	0.006±0.002^{a}	0.016±0.008^{a}	0.128±0.015^{bc}
Lactobacillaceae (Adjusted p value between C and A =0.06)	0.253±0.030^{ac}	0.079±0.020^{bc}	0.394±0.047^{a}	0.326±0.045^{a}	0.342±0.038^{a}

- End of Study – Females

Family/Group	CTR	ABT	PRE	ABT+PRE	LEAN
Peptococcaceae	0.007±0.001^{ab}	0.008±0.001^{a}	0.007±0.001^{ab}	0.009±0.001^{a}	0.004±0.000^{b}
Bifidobacteriaceae	0.001±0.000^{a}	0.000±0.000^{a}	0.000±0.000^{a}	0.000±0.000^{a}	0.000±0.000^{a}
Enterococcaceae	0.002±0.001^{a}	0.000±0.000^{b}	0.000±0.000^{b}	0.002±0.001^{a}	0.000±0.000^{b}
Verrucomicrobiaceae	0.007±0.003^{a}	0.068±0.015^{b}	0.062±0.018^{b}	0.066±0.014^{b}	0.000±0.000^{a}
Porphyromonadaceae	0.030±0.005^{a}	0.051±0.011^{a}	0.057±0.005^{a}	0.048±0.009^{a}	0.003±0.001^{b}
Enterobacteriaceae	0.001±0.000^{a}	0.001±0.000^{a}	0.002±0.001^{a}	0.003±0.001^{a}	0.001±0.000^{a}
Erysipelotrichaceae	0.029±0.006^{a}	0.007±0.002^{b}	0.048±0.008^{a}	0.009±0.002^{b}	0.054±0.025^{a}
Prevotellaceae	0.032±0.008^{ac}	0.004±0.004^{b}	0.007±0.004^{b}	0.024±0.016^{ab}	0.160±0.020^{c}
Ruminococcaceae	0.064±0.008^{a}	0.086±0.013^{a}	0.048±0.007^{a}	0.069±0.006^{a}	0.159±0.018^{b}
Peptostreptococcaceae	0.164±0.015^{ab}	0.117±0.017^{b}	0.223±0.006^{a}	0.126±0.020^{b}	0.087±0.024^{b}
Bacteroidales_S24-7_group	0.214±0.014^{a}	0.032±0.005^{b}	0.107±0.026^{bc}	0.060±0.012^{b}	0.146±0.012^{ac}
Clostridiaceae_1	0.160±0.016^{a}	0.127±0.025^{a}	0.158±0.017^{a}	0.155±0.023^{a}	0.034±0.015^{b}
Bacteroidaceae	0.012±0.002^{ac}	0.187±0.014^{b}	0.052±0.006^{cd}	0.141±0.021^{bd}	0.010±0.003^{a}
Lachnospiraceae	0.105±0.016^{a}	0.147±0.016^{a}	0.102±0.012^{a}	0.144±0.009^{a}	0.111±0.017^{a}
Lactobacillaceae	0.150±0.024^{ab}	0.151±0.035^{ab}	0.119±0.015^{a}	0.134±0.014^{ab}	0.222±0.022^{b}

Values displayed are mean±SE (relative abundance). Kruskal-Wallis test with Dunn post-hoc tests and Benjamin-Holmes False Discovery Rate (FDR) correction; $P<0.05$. CTR, control; ABT, antibiotic; PRE, prebiotic; ABT+PRE, antibiotic+prebiotic; LEAN, lean control.

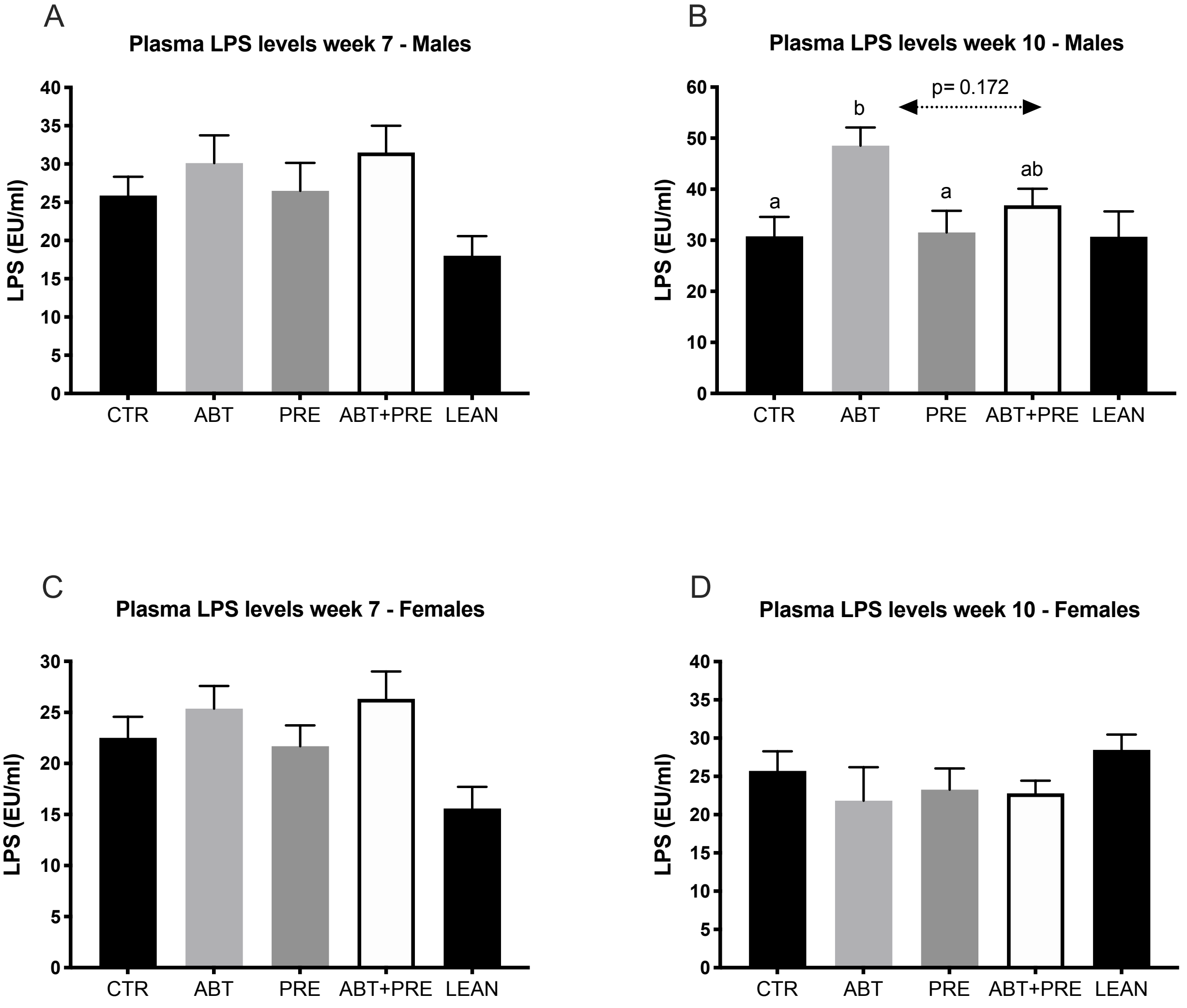

Supplemental Figure 1: **ABT male offspring display increased endotoxemia at the end of the study.** (A and B) Circulating LPS levels in males at (A) week 7 of life and (B) end of the study (n=8-10 rats per group/time point). (C and D) Circulating LPS levels in females at (C) week 7 of life and (D) end of the study (n=8-10 rats per group/time point). Different superscript letters denote significant differences between the groups, $p < 0.05$. CTR, control; ATB, antibiotic; PRE, prebiotic; ATB+PRE, antibiotic+prebiotic; LEAN, lean control.
